# Consultations for clinical features of possible cancer and associated urgent referrals before and during the COVID-19 pandemic: an observational cohort study from English primary care

**DOI:** 10.1038/s41416-021-01666-6

**Published:** 2021-12-21

**Authors:** Brian D. Nicholson, José M. Ordóñez-Mena, Sarah Lay-Flurrie, James P. Sheppard, Harshana Liyanage, Dylan McGagh, Julian Sherlock, John Williams, Margaret Smith, Cynthia Wright Drakesmith, Nicholas P. B. Thomas, Eva J. A. Morris, Rafael Perera, Simon de Lusignan, F. D. Richard Hobbs, Clare R. Bankhead

**Affiliations:** 1 Nuffield Department of Primary Care Health Sciences, University of Oxford, Oxford, UK; 2 NIHR Oxford Biomedical Research Centre, Oxford, UK; 3 UK Health Security Agency, 23 Stephenson Street Birmingham, Birmingham, B2 4BH UK; 4 Magdalen College, University of Oxford, Oxford, OX1 4AU UK; 5 Royal College of General Practitioners, 30 Euston Square, London, NW1 2FB UK; 6 Nuffield Department of Population Health, University of Oxford, Oxford, UK

**Keywords:** Epidemiology, Cancer prevention

## Abstract

**Background:**

It remains unclear to what extent reductions in urgent referrals for suspected cancer during the COVID-19 pandemic were the result of fewer patients attending primary care compared to GPs referring fewer patients.

**Methods:**

Cohort study including electronic health records data from 8,192,069 patients from 663 English practices. Weekly consultation rates, cumulative consultations and referrals were calculated for 28 clinical features from the NICE suspected cancer guidelines. Clinical feature consultation rate ratios (CRR) and urgent referral rate ratios (RRR) compared time periods in 2020 with 2019.

**Findings:**

Consultations for cancer clinical features decreased by 24.19% (95% CI: 24.04–24.34%) between 2019 and 2020, particularly in the 6–12 weeks following the first national lockdown. Urgent referrals for clinical features decreased by 10.47% (95% CI: 9.82–11.12%) between 2019 and 2020. Overall, once patients consulted with primary care, GPs urgently referred a similar or greater proportion of patients compared to previous years.

**Conclusion:**

Due to the significant fall in patients consulting with clinical features of cancer there was a lower than expected number of urgent referrals in 2020. Sustained efforts should be made throughout the pandemic to encourage the public to consult their GP with cancer clinical features.

## Introduction

Public health measures introduced to control SARS-CoV-2 transmission have altered the way patients interact with healthcare services globally [1–6]. Globally, healthcare utilisation decreased by about a third during the pandemic [7]. In England, from early March 2020, people with symptoms of COVID-19 were advised to call a national helpline instead of presenting to their general practitioner (GPs). One and a half million people considered high risk for developing severe COVID-19 were advised to ‘shield’ by staying at home for at least 12 weeks [8]. A national lockdown was introduced on March 23rd urging the population to “stay at home, protect the NHS, save lives” [9]. Clinical services almost entirely halted to develop “COVID-19 secure” ways of working to minimise the risks of nosocomial infection [3, 10, 11]. Routine appointments, procedures and non-urgent duties, in both primary and secondary care, were cancelled [12, 13]. Some of these measures were reinstated regionally after periods of relaxation due to rising rates of SARS-CoV-2 infection [9]. On October 31st a second 4 week national lockdown was introduced, in which pubs, restaurants, gyms and non-essential shops were closed but schools, colleges and universities stayed open. These restrictions have led to so called “collateral damage” to the diagnosis and management of non-COVID-19 diseases, such as cancer [14–17].

GPs in England refer patients who have clinical features (symptoms, signs, or test result abnormalities) of possible cancer meeting National Institute of Health and Care Excellence (NICE) guideline criteria for urgent cancer investigation via urgent two-week-wait (2WW) pathways [18]. Over half of all cancers are diagnosed in this way, with diagnosis in the emergency department and after non-urgent (routine) GP referral the next most common routes [19]. Typically, urgent referral pathways are organised by clinical speciality, for example, clinical features related to oesophageal, gastric, and pancreatic cancer are grouped as the upper-gastrointestinal urgent referral pathway [18]. Urgent referrals rates dropped by up to 66% in April 2020 compared to the equivalent month in 2019 with slow recovery thereafter [20]. Reductions in urgent referral rates are likely to have caused delays in cancer diagnosis but it remains unclear whether these reductions were the result of fewer patients attending primary care with clinical features of cancer or GPs referring fewer patients to hospital due to concerns about the risks associated with COVID-19 (or a combination of both) [14, 15, 21–23]. It has been hypothesised that patients with red-flag symptoms (e.g. rectal bleeding or a breast lump) would continue to present to their GP and be referred as usual [21, 24–26]. However, patients experiencing non-specific symptoms (e.g. fatigue and change in bowel habit) may be more likely to dismiss or self-manage their symptoms at home and therefore not present to their GP.

This study aimed to quantify primary care activity by week in 2020 and to compare it to previous years. Specifically, consultations for clinical features of cancer and associated cancer site specific GP urgent cancer referrals were analysed to better understand how these changes may have impacted overall rates of urgent referral.

## Methods

### Study design and population

We conducted a nationwide cohort study, utilising electronic health records from patients registered at primary care practices in England contributing to the Oxford Royal College of General Practitioners Clinical Informatics Digital Hub (ORCHID) [27]. ORCHID hosts one of Europe’s oldest sentinel practice systems, with practices recruited to be nationally representative across NHS regions, ethnicity, socio-economic status and rurality [28]. Consultation data were extracted for all registered patients of any age occurring between 1st January 2018 and 31st December 2020 with 6 weeks follow-up for urgent referrals to be identified. For patients consulting towards the end of 2020 we continued to follow-up for a further 6 weeks in order to establish whether a 2WW urgent referral was made (hereafter referred to as “urgent referral”).

Two experienced patient and public representatives commented on the protocol and the research aims. The protocol for this study was accepted by an independent approval committee and received ethical approval from the University of Oxford, Medical Sciences Interdivisional Research Ethics Committee (ref: R69874/RE001).

### Clinical features and urgent referrals

Twenty-eight symptoms, signs, and laboratory test abnormalities (hereafter referred to as “clinical features”) were selected for being included in reccomendations for the investigation of eight specific cancer sites in the 2015 NG12 NICE Suspected Cancer guideline by two clinical researchers, two researchers, and stakeholders from a national cancer charity [18]. SNOMED CT codelists were curated for each clinical feature and associated cancer specific urgent referral pathway by a clinical researcher, epidemiologist, a medical student, and two SQL developers (Supplementary Table 1). Clinical features were matched with eight selected associated cancer specific urgent referral pathways (breast, colorectal, gynaecological, haematological, head & neck, lung, upper GI and urological).

### Statistical analysis

Descriptive statistics were used to define the characteristics of the study population. Clinical feature consultation rates and urgent cancer referral rates were plotted for each week from 1st January 2018 through to 31st December 2020 to visualise pattern of consultation over time before and after the pandemic.

Rates of clinical features and urgent referrals were calculated per 100-person-years of observation, using the denominator of total registered patients that week. For breast lump, distension, and postmenopausal bleeding the denominator was women only. The denominator was men only for lower urinary tract symptoms and testicular mass or pain. Directly standardised rates for 2018 and 2019 were calculated by applying observed week-specific rates in 2018 and 2019 to the weekly denominator population in 2020, hence allowing direct comparisons of expected weekly consultation rates in 2018 and 2019 with actual rates in 2020.

Cumulative numbers of consultations for clinical features and of urgent referrals for each clinical feature-urgent referral pairing were derived for 2018, 2019 and 2020, respectively.

The cumulative sum of the expected numbers for 2018 and 2019, and actual numbers for 2020, were plotted to allow visual inspection of patterns of clinical feature reports and referrals for clinical feature-urgent referral pairings between years.

Clinical feature consultation rate ratios (CRR) with 95% confidence intervals and clinical feature-urgent cancer referral rate ratios (RRR) with 95% confidence intervals were calculated to compare rates between 2020 and 2019. A CRR greater or lower than 1 indicates a greater or lower consultation rate for a particular clinical feature, respectively, and a RRR greater or lower than 1 indicates a greater or lower referral rate for a particular clinical feature-urgent referral pairing, respectively, for the period in 2020 compared to 2019. The a priori intention had been to compare 2020 with an average of rates for 2018 and 2019 but the descriptive analysis noted changing rates of indicator and referral recording between 2018 and 2019 making the comparison to an average 2018/2019 value misleading. The a priori intention had also been for CRRs for the pre-lockdown period (weeks 1–12) and the period following the start of lockdown (weeks 13–52) to be compared with equivalent periods in previous years. It was decided post-hoc to include 6-weekly CRRs (weeks 1–6, 7–12, 13–18, 19–24, 25–30, 31–36, 37–42, 43–52) as the descriptive analysis indicated that decreases in consultations for cancer clinical features occurred prior to the date of lockdown and that recovery post-lockdown was non-linear with variation between clinical features.

The actual and percentage overall change in consultations and urgent referrals for each clinical feature-cancer site pairing were calculated comparing the 1st January to 31st December in 2020 with 2019.

## Results

### Cohort description

The cohort included 8,192,069 active patients from 663 general practices with a mean age of 38.1 ± 23.6 years, 49.3% were male, the majority were of white ethnicity (65.5% [20.9% had unknown ethnicity]), 20.9% were from the least deprived IMD quintile falling to 17.6% in the most deprived [20.9% had unknown IMD status] (Table 1). In total, there were 21,201,988 patient years (1,102,503,384 patient weeks) of observation.

**Table 1:** Characteristics of patients included in the analysis.

Patient characteristic	Total population (*n* = 8,192,069)	
	Mean/N	SD/%
Male	4,041,152	49.3%
Age (years)	38.1	23.6
Ethnic group		
White	5,367,003	65.5%
Asian	603,615	7.4%
Black	257,111	3.1%
Mixed	145,815	1.8%
Other	110,213	1.3%
Unknown	1,708,312	20.9%
IMD Quintile		
1 (most deprived)	1,443,952	17.6%
2	1,517,952	18.5%
3	1,577,455	19.3%
4	1,629,147	19.9%
5	1,713,950	20.9%
Unknown	309,613	3.8%
Urban/rural		
Urban	6,846,549	83.6%
Rural	1,316,732	16.1%

*IMD* indices of multiple deprivation.

### Overall rates of clinical feature recording and urgent cancer referral

**Fig. 1:**
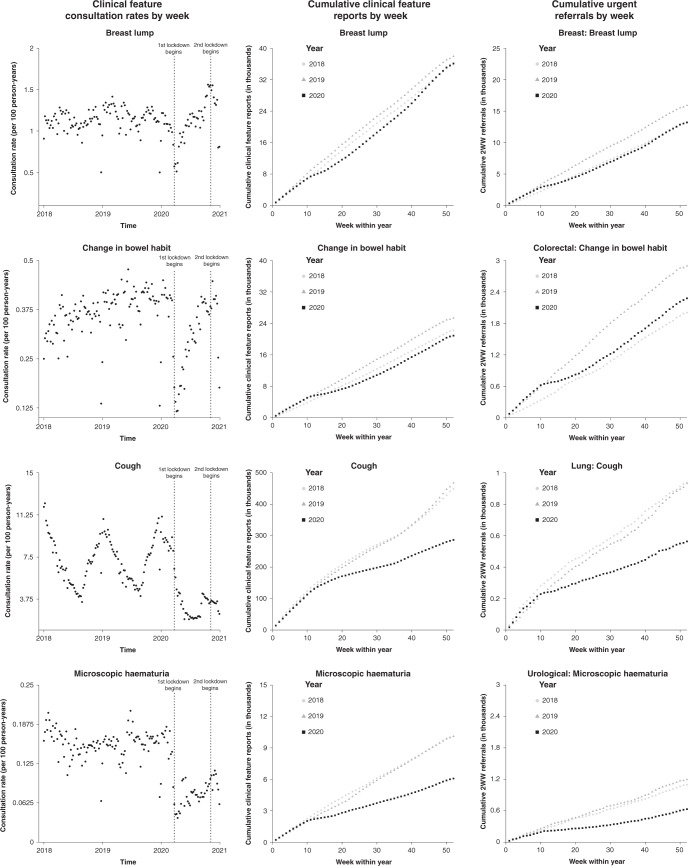
Clinical feature consultation rates by week, cumulative clinical feature reports per week (in thousands), and cumulative urgent referrals per week (in thousands) for breast lump, change in bowel habit, cough, and microscopic haematuria. These clinical features were selected to highlight disctinct patterns observed across the three panels that are considred in turn in the discussion.

Plots of weekly clinical feature recording rates showed a marked decrease at the point of the first national UK lockdown (week 13) and again around the second lockdown (week 44) (Fig. 1, Supplementary Fig. 1). There was marked variation by season in the recording of most clinical features. Cumulative plots showed that the return to the expected trajectory varied by clinical feature (Fig. 1, Supplementary Fig. 1). Overall weekly cancer specific urgent referral rates (regardless of clinical feature) also dropped for all pathways at the time of the first national lockdown (Supplementary Fig. 2). Weekly referrals to six of the urgent referral pathways showed returns to pre-lockdown rates by the end of the study period (breast, colorectal, gynaecological, haematological, head and neck, and upper-gastrointestinal) (Supplementary Fig. 2), whilst referrals to lung and urological urgent referral pathways remained lower.

### Clinical feature consultation rate ratios (CRRs)

For most (26, 90%) clinical features, consultation rates were lower than expected in the period immediately prior to lockdown (weeks 7–12) and for more than half (17, 59%) in the first six weeks of 2020 (Table 2, Supplementary Table 2) compared to the same periods in 2019. Following a significant drop after lockdown, consultations for breast lump, constipation, dysphagia, frank haematuria, jaundice, rectal bleeding, and testicular mass or pain returned to expected rates within four months of lockdown commencing (weeks 25–30) (Table 2, Supplementary Table 2). A further five symptoms returned to expected rates by the end of the year (weeks 43–52): change of bowel habit, distension, lower urinary tract symptoms, nausea, and upper abdominal pain. At this time greater than expected consultation rates were observed for breast lump, constipation, distension, rectal bleeding, and testicular mass or pain. For ten (34%) clinical features consultations remained between 0 and 20% below expected by the final period of observation; diarrhoea, haemoptysis, and microscopic haematuria remained 20% to 40% below expected; and cough, hoarse voice, and lymphadenopathy remained more than 40% less than expected (Table 2, Supplementary Table 2).

**Table 2:** Clinical feature consultation rate ratios (CRR) comparing time periods in 2020 with 2019. First national lockdown started in week 13. Legend: Increase in CRR: 1 green arrow; no change in CRR: amber arrow; 0–20% reduction in CRR: 1 red arrow; 20–40% reduction in CRR: 2 red arrows; >40% reduction in CRR: 3 red arrows. *=week 45 was the start of the second lockdown that lasted four Wks: weeks.

### Referral rate ratios (RRR) for clinical feature-urgent referral pairings

**Fig. 2:**
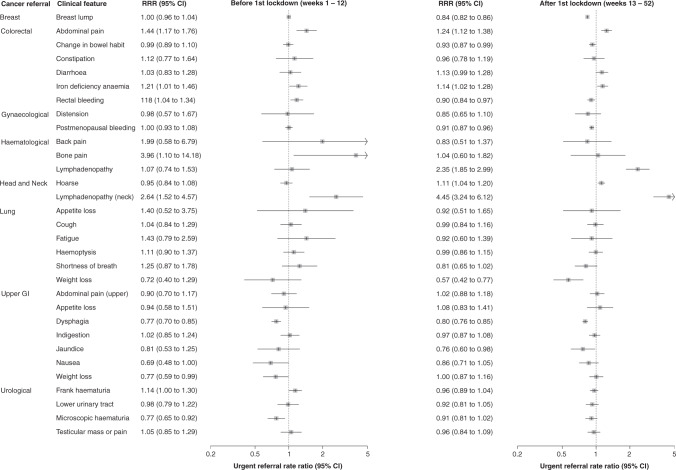
Urgent referral rate ratios (RRR) comparing the periods before and after lockdown in 2020 with respect to 2019. Clinical feature-urgent referral pairings are ordered alphabetically by the cancer site of the urgent referral and then the paired clinical feature.

**Fig. 3:**
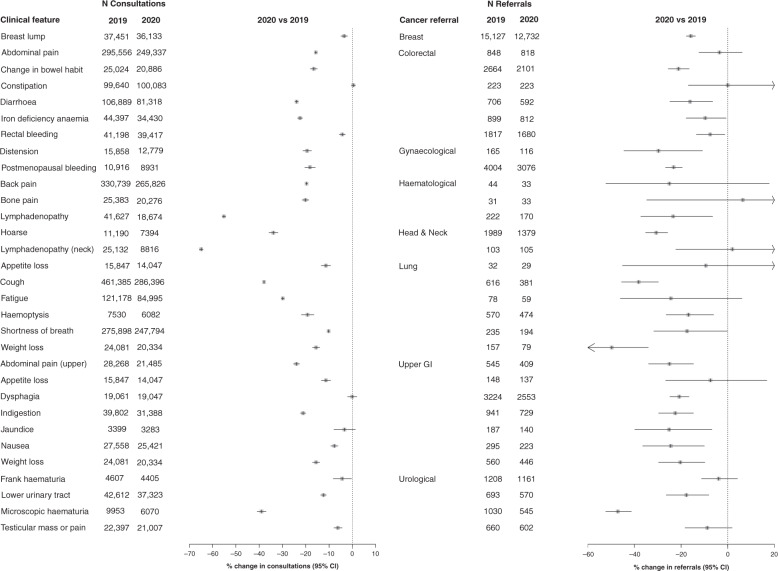
Overall reduction in consultations and associated urgent referrals for clinical features of cancer for 2020 compared to 2019. The percentage change in consultations/referrals between 2019 and 2020 was calculated as the ratio of the difference in the number of consultations/referrals between both years by the number of consultations/referrals in the reference year of 2019.

For most clinical feature-urgent referral pairings (19, 61%), GPs referred a similar proportion of patients in the period following lockdown as they did in the equivalent period of 2019 (Fig. 2). There was however a reduction in referrals for seven (23%) clinical feature-urgent referral pairings: breast lump, change in bowel habit, dysphagia, jaundice, postmenopausal bleeding, rectal bleeding, weight loss (lung) (Fig. 3). GPs referred a greater proportion of patients with abdominal pain, iron deficiency anaemia, hoarse voice, and lymphadenopathy.

### Overall changes

Overall, there was a 24.19% (95% CI: 24.04–24.34%) reduction in consultations from 2,263,439 in 2019 to 1,715,965 in 2020. This ranged from no change for dysphagia −0.07% (95%CI −2.06 to 1.95%) to a 64.92% (64.06–65.76%) decrease for lymphadenopathy of the neck (Fig. 3). There was an overall 10.47% (95% CI: 9.82–11.12%) reduction in the number of referrals for the selected clinical features from 155,220 in 2019 to 138,962 in 2020. This ranged from no change for lymphadenopathy (neck) 1.94% (−22.32 to 33.78%) to a 49.68% (34.06–61.6%) reduction for weight loss (lung).

## Discussion

### Summary of main findings

Our findings indicate the extent that fewer patients consulting with primary care about clinical features of cancer contributed to the overall reduction in English urgent cancer referrals during the first wave of the COVID-19 pandemic. Overall, once patients consulted with primary care, GPs urgently referred a similar or greater proportion of patients to what would have been expected based on data from previous years. This is illustrated by the overall reduction in consultations for clinical features being larger than the reduction in urgent referrals. As fewer patients had contacted their GP, and for some symptoms fewer referrals were made, the overall rates of urgent referral had not returned to pre-lockdown levels by the end of the study period, leaving a substantial and persistent deficit of consultations for clinical features of cancer and an associated deficit in urgent referrals compared to previous years.

### Strengths and limitations

In a large cohort of 8,192,069 people, we describe patterns of consultation and associated urgent referral activity for clinical features of cancer in the year 2020 compared to previous years. The changes in primary care activity observed between 2018 and 2019 reflect an underlying increasing trend in primary care activity [29, 30]. We show that the pandemic, in many cases, caused rates of consultation and referral to regress to 2018 levels instead of increasing further from 2019 to 2020. Insomuch our estimates of reduced activity in 2020 compared to 2019 are conservative. Using 2018 and 2019 data combined as a comparison to 2020, as initiailly intended, would have blunted the effect of the increase in activity in 2019 creating even more conservative estimates. This is the largest analysis from primary care to document the impact of the COVID-19 pandemic on consultations for clinical features and associated urgent referrals for cancer. We included no age cut-off, investigated symptoms in isolation, and restrict our analysis only by sex for sex-specific cancers. Most of the NICE guideline recommendations include a lower age limit above which referral is indicated, and some specify symptom combinations [18]. However, we have elucidated clinical feature-specific-COVID-19-signatures for the most common urgent referral pathways and in doing so provide the first quantitative evidence from primary care to underpin the interpretation of national trends in cancer referral, diagnosis, and treatment, and to inform public awareness campaigns [20, 31, 32].

The present data do not permit examination of the entire cancer diagnostic pathway from referral to subsequent clinical outcomes, such as cancer diagnosis, morbidity, and survival. Data from UK cancer registries suggested that the proportion of people diagnosed with cancer following urgent referral (the conversion rate) ranged from 1% to 20% before the pandemic depending on the urgent referral pathway [19]. Despite including a large primary care cohort of over eight million patients, the number of cancers diagnosed for most of these pairings would be too small to have confidence in the reporting of associated conversion rates. To investigate how changes in clinical feature epidemiology are linked to changes in 2020/2021 conversion rates we will extend the current cohort when enough time has passed with linkage to the National Cancer Registration and Analysis Service (NCRAS) cancer registry [33]. This longer follow-up time will allow diagnostic resolution to be reached and ensure accurate cancer registrations data for cancer site and stage to be captured. A larger dataset will increase confidence in our findings by reducing the influence of chance for some clinical feature strata with small samples in the current analysis.

Timely analysis of primary care electronic health records data was permitted due to the regular uploading into the ORCHID database [27]. Similar to other primary care databases, analyses were limited to coded data that can be subject to recording bias [34]. The switch to remote consultation during the first lockdown may have changed clinician coding behaviour. Any systematic change would have persisted as reduced clinical feature recording rates. However, following lockdown, recording rates increased for almost all clinical features investigated, and for some a return to pre-lockdown rates was observed. These observations offer reassurance that changes in clinical feature recording reflect changes in consulting patterns rather than changes in clinician coding behaviour. The coding of urgent referrals is much less prone to recording bias as it is an administrative component of the referral event. Trends in urgent referrals observed here correspond to national trends based on hospital data making it likely that these data offer a true representation of urgent referral practice [20].

### Comparison with existing literature

Globally, reports are emerging about the impact of COVID-19 on routine cancer testing, diagnostic timeliness, and the proportion of patients diagnosed with late-stage cancer [2, 4, 7, 16, 22, 35–38]. To date, the majority of English studies examining the impact of the COVID-19 pandemic have focussed on secondary care activity [14, 15, 17, 22, 39–41]. Early data showed that cancer referrals dropped by 75% [25], endoscopy procedures reduced by 95% [17], attendances at accident and emergency dropped by 35% [42]. Modelling studies have estimated the effect of the pandemic in terms of excess mortality, delayed diagnoses, avoidable cancer deaths, delayed surgery, and cancer survival [15, 16, 22, 39, 43]. There are early indications that compensatory increases in some areas of clinical activity (e.g. radiotherapy) have offset reductions in other areas (e.g. surgery) [41]. A sustained reduction in the number of people referred, diagnosed, and treated for colorectal cancer has been reported across the NHS throughout 2020 [17, 40]. These analyses have been unable to clarify the contribution of primary care activity to these trends. A small study of 47 urban general practices contributing data to the Salford Integrated Record database found the number of diagnoses of anxiety and depression, type 2 diabetes and circulatory conditions fell by 43–50% in the period between March and May 2020. However, the deficit in cancer diagnoses was not statistically significant [6]. A second retrospective cohort study of GP consultations for 123,947 patients aged 50 years and older across 21 English practices between April and July 2020 showed a 27% reduction in consultations for symptoms that could potentially indicate cancer during the first wave of the COVID-19 pandemic but did not examine trends following this or the impact on urgent cancer referrals [38]. Our current analyses are therefore very timely.

### Implications for research and practice

Our findings highlight the impact of COVID-19 on the major route to cancer diagnosis in England. They may also be help to explain changes in referrals to non-urgent (routine) pathways. Changes in patient attendance rather than GP referral behaviour were the major driver of reduced urgent referrals. Although we have focussed on the impact of national lockdown measures on consultation rates we also illustrate the impact of rising public awareness of COVID-19 on consultation rates prior to the first national lockdown: reductions that could represent increasing fear of contagion or a desire not to burden the health service. We also illustrate the lesser impact of the second national lockdown on consultations and referrals which was less restrictive, shorter, and occurred after health care providers and patients had become more accustomed to remote consultation and COVID-19 “secure” (safe) clinical practice.

The creation of drive-thru testing centres and community hubs to assess people for COVID-19 away from routine primary care practices may partly explain why records of cough, shortness of breath and haemoptysis did not recover like other clinical features during the study period [44]. A negative COVID-19 evaluation without clinical follow-up may offer false reassurance to patients with ongoing respiratory symptoms. Lung cancer diagnosis may have bypassed primary care if patients were referred from COVID-19 hubs to secondary care for assessment. For other people, fear and reluctance may have prevented contact with healthcare despite new and ongoing symptoms. During subsequent waves of the pandemic, health promotion and safety-netting should aim to ensure that persistent respiratory symptoms are not misattributed to infection or anxiety and that patients return for further assessment in primary care following negative COVID-19 testing [31, 45]. Urgent lung cancer referrals for weight loss significantly reduced following lockdown. These patients may have been redirected to Rapid Diagnostic Centres, the pathways being rolled out across the NHS to provide broad and rapid assessment of patients with non-specific symptoms [46], but this seems unlikely as rates of upper gastrointestinal urgent cancer referrals for weight loss remained unchanged from previous years. Urgent analyses are also required to understand the impact of COVID-19 on the routes to lung cancer diagnosis for each of its associated clinical features during 2020.

We hypothesised that consultations for red-flag cancer symptoms would be the first to return to expected rates after the immediate disruption of lockdown. As expected, consultation rates for breast lump, dysphagia, frank haematuria, jaundice, rectal bleeding, and testicular mass or pain were the first to return to expected levels. This suggests that patients associate these red-flag symptoms with cancer sufficiently enough not to prevent consultation with primary care, equally they are symptoms that tend to persist, interrupt day-to-day life, and may be too alarming or troublesome [47–49]. It is interesting that constipation followed a similar pattern despite not being widely regarded as a red-flag symptom. It is less clear why some other clinical features regarded to be cancer red-flags did not return to expected rates of recording as quickly, notably haemoptysis, postmenopausal bleeding, and weight loss. These symptoms may have been transitory and ignored or misattributed to changes in lifestyle and deserve further investigation or patients may have presented via other routes [50–52]. Although consultation rates returned to expected, urgent referrals for people contacting primary care with breast lump, dysphagia, jaundice, and rectal bleeding remained lower than expected in the period following lockdown. These departures from what may have been expected may be explained by patient preferences to be managed in the community, younger lower-risk patients continuing to contact their GP whilst older higher-risk patients shielded at home, or older frailer patients decided with their GPs not to investigate their symptoms when faced with the risk of COVID-19. Such modifications of referral behaviour are therefore likely to incorporate appropriate modifications of clinical judgement within the context of significant health system pressures and changes in patient preference for invasive investigation.

Consultations for gastrointestinal symptoms (abdominal pain, appetite loss, change in bowel habit, diarrhoea, distension, indigestion, nausea, upper abdominal pain), fatigue, lower urinary tract symptoms, and pain (back and bone) recovered much slower throughout the year. These are predominantly non-specific clinical features, and can be more easily normalised or attributed to changes in lifestyle (e.g. age, changes in diet, increased sedentary behaviour) [53]. In some instances, they are self-limiting after a period of (enforced) self-management with over the counter remedies or restricted access to primary care. As they do not represent well-known red flags for cancer, patients may have been more fearful of the risks of contracting COVID-19 and their symptom may have resolved before deciding to contact their GP. For these reasons, it is unlikely that there is a “reservoir” of these indicators building up that will present to primary care at a later date [24, 26]. For those patients who did contact their GP with these symptoms, the expected rates of urgent cancer referrals were observed.

The slowest recovery in clinical feature recording was observed for cough, hoarse voice, lymphadenopathy, and microscopic haematuria. The first three are also clinical features of other upper respiratory tract infections which are likely to have reduced due to suppression measures to stop the spread of SARS-CoV-2. The latter two indicators are most susceptible to reductions in face-to-face consultations [24, 54]. Lymphadenopathy might not be recorded as a neck lump on history alone, and may not be reported by the patient or detected by a clinician without clinical examination [55, 56]. Microscopic haematuria requires a urine sample to be tested at the surgery [57]. In addition to examination findings and investigations, concerns exist about whether the marked increases in remote consultation may have led to missed diagnostic cues, triggers of clinician gut feeling, and inequities in patients accessing services [24, 26, 54, 58, 59]. Due to the relatively small numbers of records for these clinical features, our findings should be cautioned, and the greatest relative increase in 2WW referral activity was observed for hoarse voice, lymphadenopathy an iron deficiency anaemia. These trends support plans to provide community diagnostic hubs to tackle rising demand for diagnostics and the backlog of patients now waiting for investigations as a result of the pandemic. If our repeat analyses in larger datasets show persistent reductions in consultations for these clinical features they may be targeted in public awareness campaigns [60].

Reductions in urgent cancer referrals may be less of a concern if the highest risk patients with qualifying clinical features are being referred. Faecal immunochemical testing (FIT) has been introduced at pace across the NHS as a non-invasive triage test for people with symptoms of colorectal cancer [39, 61, 62]. The impact of FIT and other non-invasive approaches to patient triage require evaluation in relation to cancer outcomes such as symptom-specific conversion rates, but could partly explain the reduction in urgent cancer referral seen for change in bowel habit and rectal bleeding [63, 64]. Increasing conversion rates and static detection rates may indicate that COVID-19 has introduced system efficiencies that may benefit NHS demand and patient outcomes in the long-term [17, 65].

## Conclusion

Practices and their patients are now more familiar with remote consultations, so disruptions to primary care consultations may be lesser in further waves of the pandemic. Sustained efforts should be made throughout subsequent waves of the pandemic to encourage the general public to consult their GP with symptoms of cancer. In particular, but not exclusively, for those red-flag symptoms which have not returned to expected rates: haemoptysis and unexpected weight loss. Public awareness of the availability of primary care services should also be increased and appropriate health-seeking behaviour encouraged in spite of increasing background rates of COVID-19. Within primary care systems, safety-netting methods, to ensure follow-up of patients is completed until symptoms resolve or a diagnosis is reached, should be prioritised implemented and maintained.

## Supplementary information

Supplemental Material - Supplemental tables and figures

RECORD checklist

Reproducibility checklist

## Data Availability

Permission to access the data used in this study can be obtained from the study team (https://www.phc.ox.ac.uk/research/cancer-research-group/monitoring-attendance-investigations-referrals-and-outcomes-during-covid-19-mainroute).

## Code availability

All analyses were conducted using R (version 4.0.5). The code used to perform the analysis is available on request to the authors.
