# Supplementary material for: Consultations for clinical features of possible cancer and associated urgent referrals before and during the COVID-19 pandemic: an observational cohort study from English primary care
Source: Br J Cancer. 2021 Dec 21;126(6):948–56. doi: 10.1038/s41416-021-01666-6 (PMC8691390; doi:10.1038/s41416-021-01666-6)

Supplementary Material for

**Consultations for clinical features of possible cancer and associated urgent referrals before and during the COVID-19 pandemic: an observational cohort study from English primary care.**

by Nicholson BD et al.

**Supplementary Tables**

**ST1 - SNOMED CT codelists for each clinical feature and cancer specific urgent referral pathway.**

| **Clinical feature** | **SNOMED CT code** | **Primary Term** | **Total** |
| --- | --- | --- | --- |
| Apetite loss | "206915006" | [D]Anorexia | 0 |
|  | "496871000000107" | [D]Anorexia | 7425 |
|  | "432541000000106" | [D]Anorexia NOS | 0 |
|  | "451151000000109" | [D]Anorexia NOS | 0 |
|  | "206917003" | [D]Anorexia NOS | 0 |
|  | "206916007" | [D]Appetite loss | 0 |
|  | "496881000000109" | [D]Appetite loss | 0 |
|  | "249468005" | Anorexia symptom | 30568 |
|  | "269813009" | Appetite loss - anorexia | 0 |
|  | "249470001" | Cannot face food | 19 |
|  | "64379006" | Decrease in appetite | 67003 |
|  | "39161000000101" | Decrease in appetite | 0 |
|  | "41421000000102" | Decrease in appetite | 0 |
|  | "46641000000101" | Decrease in appetite | 0 |
|  | "78901000000106" | Decrease in appetite | 0 |
|  | "79890006" | Loss of appetite | 44744 |
|  | "249471002" | Loss of appetite - symptom | 0 |
|  | "249469002" | No interest in food | 136 |
| Change in bowel habit | "207147008" | [D]Change in bowel habit | 0 |
|  | "498661000000103" | [D]Change in bowel habit | 0 |
|  | "88111009" | Altered bowel function | 313043 |
|  | "139374009" | Altered bowel function | 0 |
| Constipation | "191971009" | (Psychogenic diarrhoea) or (spurious diarrhoea) | 5 |
|  | "197119006" | Acute constipation | 8191 |
|  | "58230007" | Alteration in bowel elimination: constipation | 1 |
|  | "432414001" | Atonic constipation | 0 |
|  | "707831000000103" | Chronic constipation | 0 |
|  | "1726521000006115" | Chronic constipation | 1021 |
|  | "236069009" | Chronic constipation | 18398 |
|  | "31499008" | Chronic constipation with overflow | 13460 |
|  | "197120000" | Chronic constipation without overflow | 3953 |
|  | "82934008" | Chronic idiopathic constipation | 2 |
|  | "14760008" | Constipation | 1626457 |
|  | "249517009" | Constipation alternates with diarrhoea | 194 |
|  | "85920003" | Constipation by outlet obstruction | 0 |
|  | "432994008" | Constipation due to neurogenic bowel | 0 |
|  | "430097009" | Constipation due to spasm of colon | 0 |
|  | "418013002" | Defaecation reflex abnormal - constipated | 0 |
|  | "21782001" | Drug-induced constipation | 4186 |
|  | "73149003" | Encopresis with constipation AND overflow incontinence | 0 |
|  | "45925007" | Enema for removal of impacted faeces | 0 |
|  | "44635007" | Faecal impaction | 7067 |
|  | "29162007" | Faecal impaction of colon | 0 |
|  | "313280002" | Manual evacuation of faeces from rectum | 1851 |
|  | "174297001" | Manual removal of impacted faeces from rectum | 363 |
|  | "163329000" | O/E - PR-rectum full of faeces | 1961 |
|  | "140539001" | O/E - PR-rectum full of faeces | 0 |
|  | "111360009" | Obstipation | 0 |
|  | "163890000" | On examination - defaecation reflex abnormal - constipated | 295 |
|  | "660811000000104" | Other specified constipation | 0 |
|  | "129585003" | Perceived constipation | 0 |
|  | "1339004" | Removal of impacted faeces | 3488 |
|  | "236070005" | Simple constipation | 1401 |
|  | "35298007" | Slow transit constipation | 271 |
|  | "136801000119102" | Therapeutic opioid induced constipation | 0 |
| Distension | "498531000000107" | [D]Abdominal distension, gaseous | 0 |
|  | "158435003" | [D]Abdominal distension, gaseous | 0 |
|  | "207128005" | [D]Abdominal distension, gaseous | 0 |
|  | "207238007" | [D]Abdominal swelling | 0 |
|  | "158434004" | [D]Abdominal swelling | 0 |
|  | "499341000000103" | [D]Abdominal swelling | 0 |
|  | "498541000000103" | [D]Bloating | 0 |
|  | "158436002" | [D]Bloating | 0 |
|  | "207129002" | [D]Bloating | 0 |
|  | "158437006" | [D]Tympanites (abdominal) | 0 |
|  | "139348003" | Abd. distension symptom NOS | 0 |
|  | "139345000" | Abdomen feels bloated | 0 |
|  | "162070003" | Abdomen feels bloated | 0 |
|  | "162071004" | Abdomen feels distended | 0 |
|  | "139346004" | Abdomen feels distended | 0 |
|  | "139347008" | Abdomen feels swollen | 0 |
|  | "162072006" | Abdomen feels swollen | 0 |
|  | "116289008" | Abdominal bloating | 7 |
|  | "41931001" | Abdominal distension | 5 |
|  | "162068007" | Abdominal distension symptom | 36799 |
|  | "139343007" | Abdominal distension symptom | 0 |
|  | "162073001" | Abdominal distension symptom NOS | 0 |
|  | "583831000000109" | Abdominal distension symptom NOS | 0 |
|  | "271835004" | Abdominal distension, gaseous | 2072 |
|  | "84454003" | Abdominal mass | 0 |
|  | "226284003" | Abdominal swelling | 0 |
|  | "248490000" | Bloating symptom | 251354 |
|  | "162074007" | Flatulence &/or wind (& [symptom: [belching] or [bloating] or [eructation] or [flatulence] or [wind]]) | 62 |
|  | "139349006" | Flatulence &/or wind (& [symptom: [belching] or [bloating] or [eructation] or [flatulence] or [wind]]) | 0 |
|  | "163203002" | On examination - uniform abdominal swelling | 106 |
|  | "60728008" | Swollen abdomen | 17477 |
|  | "300402000" | Uniform abdominal distention | 11 |
| Dysphagia | "207122006" | [D]Difficulty in swallowing | 0 |
|  | "498481000000105" | [D]Difficulty in swallowing | 0 |
|  | "498471000000108" | [D]Dysphagia | 0 |
|  | "207121004" | [D]Dysphagia | 0 |
|  | "207123001" | [D]Dysphagia NOS | 0 |
|  | "438501000000105" | [D]Dysphagia NOS | 0 |
|  | "403081000000105" | [D]Dysphagia NOS | 0 |
|  | "311808009" | Aberrant retro-esophageal subclavian artery causing dysphagia | 22 |
|  | "196841000000100" | Acquired dysphagia | 0 |
|  | "898751000000102" | Acquired swallowing difficulty | 16 |
|  | "990391000000103" | Chokes when swallowing | 0 |
|  | "225589000" | Chokes when swallowing | 940 |
|  | "65191003" | Constant low-grade dysphagia | 0 |
|  | "288967003" | Difficulty initiating swallowing reflex | 10 |
|  | "288939007" | Difficulty swallowing | 2388 |
|  | "162025006" | Difficulty swallowing fluid | 1462 |
|  | "288945004" | Difficulty swallowing food | 437 |
|  | "288962009" | Difficulty swallowing saliva | 18 |
|  | "306778008" | Difficulty swallowing soft foods | 6 |
|  | "162024005" | Difficulty swallowing solids | 7709 |
|  | "288956004" | Difficulty swallowing thickened fluid | 10 |
|  | "40739000" | Dysphagia | 178364 |
|  | "439576009" | Fear of drinking liquids due to dysphagia | 0 |
|  | "249485007" | Food sticks on swallowing | 3 |
|  | "722875003" | Functional dysphagia | 0 |
|  | "19597002" | Intermittent dysphagia | 1 |
|  | "40890009" | Oesophageal dysphagia | 4 |
|  | "429975007" | Oral phase dysphagia | 1 |
|  | "71457002" | Oropharyngeal dysphagia | 30 |
|  | "21101000119105" | Pharyngeal dysphagia | 3 |
|  | "30233002" | Swallowing painful | 16638 |
|  | "139293008" | Swallowing painful | 0 |
|  | "86361002" | Swallowing problem | 0 |
|  | "249487004" | Unable to initiate swallowing | 2 |
|  | "249486008" | Unable to swallow | 92 |
|  | "288948002" | Unable to swallow fluid | 7 |
|  | "288942001" | Unable to swallow food | 19 |
|  | "288959006" | Unable to swallow saliva | 7 |
|  | "306775006" | Unable to swallow soft foods | 2 |
|  | "306770001" | Unable to swallow solids | 8 |
|  | "288953007" | Unable to swallow thickened fluid | 5 |
|  | "398999006" | Uncompensated swallowing impairment | 0 |
| Jaundice | "155832005" | (Biliary tract disorders NOS) or (obstructive jaundice NOS) | 0 |
|  | "266545005" | (Biliary tract disorders NOS) or (obstructive jaundice NOS) | 0 |
|  | "206896007" | [D]Cholaemia NOS | 0 |
|  | "428801000000106" | [D]Cholaemia NOS | 0 |
|  | "502291000000102" | [D]Icterus | 0 |
|  | "274245007" | [D]Icterus | 0 |
|  | "206897003" | [D]Icterus NOS | 0 |
|  | "428811000000108" | [D]Icterus NOS | 1 |
|  | "496751000000106" | [D]Jaundice (not of newborn) | 0 |
|  | "206895006" | [D]Jaundice (not of newborn) | 0 |
|  | "206898008" | [D]Jaundice (not of newborn) NOS | 0 |
|  | "442291000000104" | [D]Jaundice (not of newborn) NOS | 0 |
|  | "391071000000104" | [D]Jaundice (not of newborn) NOS | 0 |
|  | "15770003" | Acute cholestatic jaundice syndrome | 1 |
|  | "44018007" | Cholestatic jaundice syndrome | 0 |
|  | "75066002" | Chronic cholestatic jaundice syndrome | 0 |
|  | "138805009" | H/O: jaundice | 0 |
|  | "161536006" | H/O: jaundice | 4421 |
|  | "18165001" | Jaundice | 51825 |
|  | "248214007" | Jaundiced appearance of face | 17 |
|  | "162740002" | O/E - jaundiced colour | 6152 |
|  | "59848001" | Obstructive hyperbilirubinaemia | 8466 |
|  | "197452009" | Obstructive jaundice NOS | 0 |
|  | "589441000000100" | Obstructive jaundice NOS | 0 |
|  | "246975001" | Scleral icterus | 21 |
|  | "161866005" | Yellow &/or jaundiced colour (& [symptom]) | 309 |
|  | "139122003" | Yellow &/or jaundiced colour (& [symptom]) | 0 |
|  | "267030001" | Yellow or jaundiced colour | 4803 |
| Breast lump | "89164003" | Breast lump | 403313 |
|  | "197981000000104" | Breast lump detected by clinician examination | 1240 |
|  | "212811000000100" | Breast lump detected by clinician examination | 0 |
|  | "212821000000106" | Breast lump detected by clinician examination | 0 |
|  | "212851000000101" | Breast lump detected by partner | 0 |
|  | "212861000000103" | Breast lump detected by partner | 0 |
|  | "198001000000106" | Breast lump detected by partner | 94 |
|  | "198011000000108" | Breast lump detected by self-examination | 5971 |
|  | "212871000000105" | Breast lump detected by self-examination | 0 |
|  | "212881000000107" | Breast lump detected by self-examination | 0 |
|  | "10750111000119108" | Breast lump in pregnancy | 0 |
|  | "139442001" | Breast lump present | 0 |
|  | "162162006" | Breast lump present | 129172 |
|  | "162160003" | Breast lump symptom | 236640 |
|  | "563471000000105" | Breast lump symptom NOS | 0 |
|  | "827144003" | Cystic lump of breast | 0 |
|  | "27431007" | Fibrocystic disease of breast | 29159 |
|  | "816059002" | Fixed deep lump of breast | 0 |
|  | "792891005" | Hard lump of breast | 0 |
|  | "792889002" | Irregular lump of breast | 0 |
|  | "792890006" | Lump of axillary tail of breast | 0 |
|  | "816058005" | Lump of breast fixed to skin | 0 |
|  | "816053001" | Lump of lower inner quadrant of breast | 0 |
|  | "816054007" | Lump of lower outer quadrant of breast | 0 |
|  | "16836091000119107" | Lump of subareolar area of left breast | 0 |
|  | "16837031000119106" | Lump of subareolar area of right breast | 0 |
|  | "816052006" | Lump of upper inner quadrant of breast | 0 |
|  | "816055008" | Lump of upper outer quadrant of breast | 0 |
|  | "653801000000102" | Lump, breast - NOS | 0 |
|  | "816056009" | Mass of nipple of breast | 0 |
|  | "816060007" | Mobile lump of breast | 0 |
|  | "163472005" | O/E-breast lump- axillary tail | 497 |
|  | "163479001" | O/E-breast lump-grapefruit size | 4 |
|  | "163471003" | O/E-breast lump-lower out-quad | 218 |
|  | "163467001" | O/E-breast lump-nipple/central | 704 |
|  | "163477004" | O/E-breast lump-tangerine size | 16 |
|  | "163468006" | O/E-breast lump-upper in-quad | 298 |
|  | "163470002" | O/E-breast lump-upper out-quad | 979 |
|  | "163490006" | O/E-breast lump regularity NOS | 0 |
|  | "140698004" | O/E-breast lump regularity NOS | 0 |
|  | "579461000000105" | O/E-breast lump regularity NOS | 0 |
|  | "163478009" | O/E - breast lump-orange size | 11 |
|  | "163480003" | O/E - breast lump - melon size | 4 |
|  | "163475007" | O/E - breast lump - pea size | 1072 |
|  | "163476008" | O/E - breast lump - plum size | 286 |
|  | "163486009" | O/E - breast lump consist. NOS | 0 |
|  | "140694002" | O/E - breast lump consist. NOS | 0 |
|  | "534201000000105" | O/E - breast lump consist. NOS | 0 |
|  | "140689002" | O/E - breast lump consistency | 0 |
|  | "163482006" | O/E - breast lump consistency | 282 |
|  | "163484007" | O/E - breast lump cystic | 2037 |
|  | "140691005" | O/E - breast lump cystic | 0 |
|  | "163494002" | O/E - breast lump fixed deep | 12 |
|  | "163485008" | O/E - breast lump hard | 350 |
|  | "163489002" | O/E - breast lump irregular | 116 |
|  | "163492003" | O/E - breast lump not tethered | 199 |
|  | "140700008" | O/E - breast lump not tethered | 0 |
|  | "268951004" | O/E - breast lump palpated | 2208 |
|  | "553691000000103" | O/E - breast lump palpated NOS | 0 |
|  | "275964000" | O/E - breast lump position | 438 |
|  | "163487000" | O/E - breast lump regularity | 105 |
|  | "140695001" | O/E - breast lump regularity | 0 |
|  | "163474006" | O/E - breast lump size | 665 |
|  | "534191000000108" | O/E - breast lump size NOS | 0 |
|  | "163488005" | O/E - breast lump smooth | 245 |
|  | "140696000" | O/E - breast lump smooth | 0 |
|  | "140690006" | O/E - breast lump soft | 0 |
|  | "163483001" | O/E - breast lump soft | 236 |
|  | "163495001" | O/E - breast lump tethered NOS | 0 |
|  | "140703005" | O/E - breast lump tethered NOS | 0 |
|  | "570951000000105" | O/E - breast lump tethered NOS | 0 |
|  | "140699007" | O/E - breast lump tethering | 0 |
|  | "163491005" | O/E - breast lump tethering | 116 |
|  | "163469003" | O/E -breast lump-lower in-quad | 173 |
|  | "163493008" | O/E -breast lump fixed to skin | 131 |
|  | "315249005" | Persistent breast nodularity | 911 |
|  | "792888005" | Smooth lump of breast | 0 |
|  | "792887000" | Soft lump of breast | 0 |
|  | "816057000" | Tethering of lump of breast | 0 |
| Lymphadenopathy | "497851000000101" | [D]Generalised enlarged lymph nodes | 0 |
|  | "207037007" | [D]Generalised enlarged lymph nodes | 0 |
|  | "207038002" | [D]Localised enlarged lymph nodes | 0 |
|  | "497861000000103" | [D]Localised enlarged lymph nodes | 0 |
|  | "497821000000106" | [D]Lymph node enlargement | 1 |
|  | "207033006" | [D]Lymph node enlargement | 0 |
|  | "158361000" | [D]Lymph node enlargement | 0 |
|  | "158364008" | [D]Lymph node enlargement NOS | 0 |
|  | "207039005" | [D]Lymph node enlargement NOS | 0 |
|  | "463481000000101" | [D]Lymph node enlargement NOS | 1 |
|  | "415111000000104" | [D]Lymph node enlargement NOS | 0 |
|  | "497831000000108" | [D]Lymphadenopathy | 5 |
|  | "207034000" | [D]Lymphadenopathy | 0 |
|  | "158362007" | [D]Lymphadenopathy | 0 |
|  | "158363002" | [D]Swollen glands | 0 |
|  | "207035004" | [D]Swollen glands | 0 |
|  | "497841000000104" | [D]Swollen glands | 0 |
|  | "425661000000104" | [X]Acute lymphadenitis of other sites | 0 |
|  | "238405002" | Acute cervical adenitis | 6170 |
|  | "583771000000101" | Acute febrile mucocutaneous lymph node syndrome NOS | 0 |
|  | "41174002" | Acute lymphadenitis | 23954 |
|  | "10629071000119102" | Acute lymphadenitis of axilla | 0 |
|  | "10629111000119109" | Acute lymphadenitis of inguinal lymph nodes | 0 |
|  | "200699009" | Acute lymphadenitis of lower limb | 168 |
|  | "200698001" | Acute lymphadenitis of upper limb | 159 |
|  | "127078001" | Anterior auricular lymphadenopathy | 1 |
|  | "127090004" | Anterior cervical lymphadenopathy | 22 |
|  | "127211005" | Anterior tibial lymphadenopathy | 0 |
|  | "127192009" | Apical axillary lymphadenopathy | 0 |
|  | "127189005" | Axillary lymphadenopathy | 404 |
|  | "302061009" | Brachial lymphadenopathy | 4 |
|  | "127191002" | Central axillary lymphadenopathy | 0 |
|  | "240414006" | Cervical atypical mycobacterial lymphadenitis | 8 |
|  | "3502005" | Cervical lymphadenitis | 1 |
|  | "127086001" | Cervical lymphadenopathy | 255782 |
|  | "127196007" | Cubital lymphadenopathy | 0 |
|  | "127200002" | Deep inguinal lymphadenopathy | 0 |
|  | "127075003" | Deep lymphadenopathy | 0 |
|  | "127207004" | Deep popliteal lymphadenopathy | 0 |
|  | "127093002" | Delphian lymphadenopathy | 0 |
|  | "274740001" | Enlarged submandibular lymph gland | 1378 |
|  | "127197003" | Epitrochlear lymphadenopathy | 0 |
|  | "127081006" | Facial lymphadenopathy | 0 |
|  | "447803000" | Femoral lymphadenopathy | 0 |
|  | "127212003" | Fibular lymphadenopathy | 0 |
|  | "274741002" | Generalised enlarged lymph nodes | 332 |
|  | "127174001" | Gluteal lymphadenopathy | 0 |
|  | "704281009" | Head and neck lymphadenopathy | 0 |
|  | "239701000000105" | Head and neck lymphadenopathy | 0 |
|  | "127159003" | Iliac lymphadenopathy | 0 |
|  | "127079009" | Inferior auricular lymphadenopathy | 0 |
|  | "127175000" | Inferior gluteal lymphadenopathy | 0 |
|  | "127205007" | Inferior inguinal lymphadenopathy | 0 |
|  | "127095009" | Infraclavicular lymphadenopathy | 0 |
|  | "127199000" | Inguinal lymphadenopathy | 280 |
|  | "127089008" | Jugular lymphadenopathy | 0 |
|  | "75053002" | Kawasaki disease | 3043 |
|  | "127190001" | Lateral axillary lymphadenopathy | 0 |
|  | "127087005" | Lateral cervical lymphadenopathy | 0 |
|  | "274744005" | Localised enlarged lymph nodes | 2933 |
|  | "127198008" | Lower extremity lymphadenopathy | 0 |
|  | "141342003" | Lymph nodes: [O/E - lymphadenopathy] or [enlarged] or [O/E] | 0 |
|  | "269044004" | Lymph nodes: [O/E - lymphadenopathy] or [enlarged] or [O/E] | 2 |
|  | "271821001" | Lymphadenopathy | 0 |
|  | "139134006" | Lymphadenopathy | 0 |
|  | "161878009" | Lymphadenopathy | 0 |
|  | "30746006" | Lymphadenopathy | 266849 |
|  | "425061006" | Lymphadenopathy of head AND/OR neck | 0 |
|  | "127084003" | Mandibular lymphadenopathy | 0 |
|  | "164148003" | O/E-sub-mental lymphadenopathy | 2789 |
|  | "141346000" | O/E-supraclav.lymphadenopathy | 0 |
|  | "164151005" | O/E - inguinal lymphadenopathy | 8952 |
|  | "164158004" | O/E - lymph nodes discrete | 577 |
|  | "164155001" | O/E - lymph nodes firm | 197 |
|  | "164159007" | O/E - lymph nodes fluctuant | 23 |
|  | "164156000" | O/E - lymph nodes hard | 26 |
|  | "164157009" | O/E - lymph nodes tethered | 2 |
|  | "164145000" | O/E - lymphadenopathy | 0 |
|  | "164153008" | O/E - lymphadenopathy NOS | 0 |
|  | "141350007" | O/E - lymphadenopathy NOS | 0 |
|  | "587001000000103" | O/E - lymphadenopathy NOS | 0 |
|  | "392783003" | O/E - post-auricular lymphadenopathy | 0 |
|  | "393737000" | O/E - post-auricular lymphadenopathy | 0 |
|  | "394677008" | O/E - post-auricular lymphadenopathy | 2551 |
|  | "164150006" | O/E -axillary lymphadenopathy | 6894 |
|  | "164147008" | O/E -cervical lymphadenopathy | 1 |
|  | "164152003" | O/E -popliteal lymphadenopathy | 33 |
|  | "127076002" | Occipital lymphadenopathy | 0 |
|  | "164149006" | On examination - supraclavicular lymphadenopathy | 994 |
|  | "127195006" | Paramammary lymphadenopathy | 0 |
|  | "127193004" | Pectoral axillary lymphadenopathy | 0 |
|  | "127206008" | Popliteal lymphadenopathy | 0 |
|  | "127077006" | Posterior auricular lymphadenopathy | 0 |
|  | "127210006" | Posterior tibial lymphadenopathy | 0 |
|  | "127201003" | Prefemoral lymphadenopathy | 0 |
|  | "127092007" | Prelaryngeal lymphadenopathy | 0 |
|  | "127094008" | Pretracheal lymphadenopathy | 0 |
|  | "445718006" | Reactive lymphadenopathy | 7146 |
|  | "725531000000101" | Reactive lymphadenopathy | 0 |
|  | "757021000000101" | Reactive lymphadenopathy | 0 |
|  | "127091000" | Retropharyngeal lymphadenopathy | 0 |
|  | "127096005" | Scalene lymphadenopathy | 0 |
|  | "15170009" | Submandibular lymphadenitis | 147 |
|  | "127082004" | Submandibular lymphadenopathy | 0 |
|  | "127083009" | Submental lymphadenopathy | 0 |
|  | "127194005" | Subscapular axillary lymphadenopathy | 0 |
|  | "127202005" | Superficial inguinal lymphadenopathy | 0 |
|  | "127208009" | Superficial popliteal lymphadenopathy | 0 |
|  | "127176004" | Superior gluteal lymphadenopathy | 0 |
|  | "127204006" | Superior lateral inguinal lymphadenopathy | 0 |
|  | "127203000" | Superior medial inguinal lymphadenopathy | 0 |
|  | "48573006" | Suppurative lymphadenopathy | 0 |
|  | "127088000" | Supraclavicular lymphadenopathy | 2 |
|  | "127209001" | Tibial lymphadenopathy | 0 |
|  | "300929003" | Tonsillar adenitis | 60 |
|  | "127188002" | Upper extremity lymphadenopathy | 0 |
|  | "76461007" | Virchow's node | 0 |
|  | "332457007" | Virchow's node | 0 |
| Lymphadenopathy (neck) | "238405002" | Acute cervical adenitis | 6170 |
|  | "583771000000101" | Acute febrile mucocutaneous lymph node syndrome NOS | 0 |
|  | "200700005" | Acute lymphadenitis of face, head and neck | 3102 |
|  | "10629031000119100" | Acute lymphadenitis of neck | 0 |
|  | "200696002" | Acute: [lymphadenitis] or [abscess lymph node] or [cervical adenitis] | 26 |
|  | "127090004" | Anterior cervical lymphadenopathy | 22 |
|  | "3502005" | Cervical lymphadenitis | 1 |
|  | "127086001" | Cervical lymphadenopathy | 255782 |
|  | "127093002" | Delphian lymphadenopathy | 0 |
|  | "274740001" | Enlarged submandibular lymph gland | 1378 |
|  | "239701000000105" | Head and neck lymphadenopathy | 0 |
|  | "704281009" | Head and neck lymphadenopathy | 0 |
|  | "127079009" | Inferior auricular lymphadenopathy | 0 |
|  | "127095009" | Infraclavicular lymphadenopathy | 0 |
|  | "127089008" | Jugular lymphadenopathy | 0 |
|  | "75053002" | Kawasaki disease | 3043 |
|  | "127087005" | Lateral cervical lymphadenopathy | 0 |
|  | "164148003" | O/E-sub-mental lymphadenopathy | 2789 |
|  | "141345001" | O/E-sub-mental lymphadenopathy | 0 |
|  | "141346000" | O/E-supraclav.lymphadenopathy | 0 |
|  | "394677008" | O/E - post-auricular lymphadenopathy | 2551 |
|  | "393737000" | O/E - post-auricular lymphadenopathy | 0 |
|  | "392783003" | O/E - post-auricular lymphadenopathy | 0 |
|  | "164147008" | O/E -cervical lymphadenopathy | 1 |
|  | "127076002" | Occipital lymphadenopathy | 0 |
|  | "164149006" | On examination - supraclavicular lymphadenopathy | 994 |
|  | "127077006" | Posterior auricular lymphadenopathy | 0 |
|  | "127092007" | Prelaryngeal lymphadenopathy | 0 |
|  | "127094008" | Pretracheal lymphadenopathy | 0 |
|  | "127091000" | Retropharyngeal lymphadenopathy | 0 |
|  | "127096005" | Scalene lymphadenopathy | 0 |
|  | "15170009" | Submandibular lymphadenitis | 147 |
|  | "127082004" | Submandibular lymphadenopathy | 0 |
|  | "127088000" | Supraclavicular lymphadenopathy | 2 |
|  | "300929003" | Tonsillar adenitis | 60 |
| Nausea | "207111007" | [D]Nausea | 0 |
|  | "158421000" | [D]Nausea | 0 |
|  | "498401000000100" | [D]Nausea | 3 |
|  | "498391000000103" | [D]Nausea and vomiting | 0 |
|  | "207110008" | [D]Nausea and vomiting | 0 |
|  | "207116002" | [D]Nausea and vomiting NOS | 0 |
|  | "425721000000101" | [D]Nausea and vomiting NOS | 0 |
|  | "400471000000109" | [D]Nausea and vomiting NOS | 0 |
|  | "300576004" | Finding of nausea | 0 |
|  | "73335002" | Increased nausea and vomiting | 0 |
|  | "698861005" | Intractable nausea and vomiting | 0 |
|  | "422587007" | Nausea | 391572 |
|  | "73879007" | Nausea | 0 |
|  | "139330007" | Nausea | 0 |
|  | "162055004" | Nausea | 0 |
|  | "272043005" | Nausea | 0 |
|  | "16932000" | Nausea and vomiting | 41578 |
|  | "162060000" | Nausea NOS | 0 |
|  | "139334003" | Nausea NOS | 0 |
|  | "582741000000100" | Nausea NOS | 0 |
|  | "139332004" | Nausea present | 0 |
|  | "162057007" | Nausea present | 37452 |
|  | "2919008" | Nausea, vomiting and diarrhoea | 1 |
| Nocturia | "207178007" | [D]Nocturia | 0 |
|  | "498911000000108" | [D]Nocturia | 0 |
|  | "139394000" | Nocturia | 179698 |
|  | "6408001" | Nocturia - finding | 0 |
|  | "1711000119101" | Nocturia due to benign prostatic hypertrophy | 0 |
| Abdominal pain | "307724003" | [D] Left upper quadrant pain | 0 |
|  | "307722004" | [D] Right upper quadrant pain | 0 |
|  | "307725002" | [D] Upper abdominal pain | 0 |
|  | "207207006" | [D]Abdominal colic | 0 |
|  | "499131000000109" | [D]Abdominal colic | 0 |
|  | "499151000000102" | [D]Abdominal cramps | 0 |
|  | "207210004" | [D]Abdominal cramps | 0 |
|  | "158504006" | [D]Abdominal cramps | 0 |
|  | "158498003" | [D]Abdominal pain | 0 |
|  | "207205003" | [D]Abdominal pain | 0 |
|  | "499111000000101" | [D]Abdominal pain | 24 |
|  | "444651000000101" | [D]Abdominal pain NOS | 0 |
|  | "390251000000109" | [D]Abdominal pain NOS | 1 |
|  | "207230000" | [D]Abdominal pain NOS | 0 |
|  | "158512003" | [D]Abdominal pain NOS | 0 |
|  | "207251002" | [D]Abdominal rigidity | 0 |
|  | "499451000000107" | [D]Abdominal rigidity | 0 |
|  | "499121000000107" | [D]Abdominal tenderness | 0 |
|  | "207206002" | [D]Abdominal tenderness | 0 |
|  | "207221008" | [D]Acute abdomen | 0 |
|  | "207255006" | [D]Acute abdomen | 0 |
|  | "158499006" | [D]Acute abdomen | 0 |
|  | "499261000000106" | [D]Acute abdomen | 0 |
|  | "274256007" | [D]Colic | 0 |
|  | "207211000" | [D]Epigastric pain | 0 |
|  | "499161000000104" | [D]Epigastric pain | 0 |
|  | "498491000000107" | [D]Flatulence, eructation and gas pain | 0 |
|  | "207124007" | [D]Flatulence, eructation and gas pain | 0 |
|  | "207131006" | [D]Flatulence, eructation and gas pain NOS | 0 |
|  | "462691000000108" | [D]Flatulence, eructation and gas pain NOS | 0 |
|  | "498521000000105" | [D]Gas pain (abdominal) | 0 |
|  | "207127000" | [D]Gas pain (abdominal) | 0 |
|  | "207213002" | [D]Hypochondrial pain | 0 |
|  | "499181000000108" | [D]Hypochondrial pain | 0 |
|  | "502821000000101" | [D]Left lower quadrant pain | 0 |
|  | "311811005" | [D]Left lower quadrant pain | 0 |
|  | "502581000000102" | [D]Left upper quadrant pain | 0 |
|  | "499231000000101" | [D]Loin pain | 0 |
|  | "207218006" | [D]Loin pain | 0 |
|  | "207228002" | [D]Nonspecific abdominal pain | 0 |
|  | "502841000000108" | [D]Nonspecific abdominal pain | 6 |
|  | "311813008" | [D]Nonspecific abdominal pain | 0 |
|  | "499281000000102" | [D]Other specified abdominal pain | 0 |
|  | "207229005" | [D]Other specified abdominal pain | 0 |
|  | "207216005" | [D]Pain in left iliac fossa | 0 |
|  | "499211000000109" | [D]Pain in left iliac fossa | 0 |
|  | "499201000000107" | [D]Pain in right iliac fossa | 0 |
|  | "207215009" | [D]Pain in right iliac fossa | 0 |
|  | "207220009" | [D]Recurrent acute abdominal pain | 0 |
|  | "499251000000108" | [D]Recurrent acute abdominal pain | 1 |
|  | "502831000000104" | [D]Right lower quadrant pain | 0 |
|  | "311812003" | [D]Right lower quadrant pain | 0 |
|  | "502571000000104" | [D]Right upper quadrant pain | 1 |
|  | "499191000000105" | [D]Suprapubic pain | 0 |
|  | "207214008" | [D]Suprapubic pain | 0 |
|  | "207212007" | [D]Umbilical pain | 0 |
|  | "499171000000106" | [D]Umbilical pain | 0 |
|  | "502591000000100" | [D]Upper abdominal pain | 1 |
|  | "462851000000101" | [X]Pain localised to other parts of lower abdomen | 0 |
|  | "207588000" | [X]Pain localised to other parts of lower abdomen | 0 |
|  | "269021002" | Abdomen: [O/E - board like rigidity] or [acute] | 0 |
|  | "140460009" | Abdomen: [O/E - board like rigidity] or [acute] | 0 |
|  | "9991008" | Abdominal colic | 143264 |
|  | "137891000119105" | Abdominal colic in adult or child greater than 12 months | 0 |
|  | "28221000119103" | Abdominal muscle pain | 0 |
|  | "21522001" | Abdominal pain | 3208420 |
|  | "139313005" | Abdominal pain | 0 |
|  | "314212008" | Abdominal pain - cause unknown | 942 |
|  | "314041007" | Abdominal pain in early pregnancy | 787 |
|  | "309737007" | Abdominal pain in pregnancy | 8976 |
|  | "199150000" | Abdominal pain in pregnancy | 0 |
|  | "74704000" | Abdominal pain through to back | 0 |
|  | "71850005" | Abdominal pain worse on motion | 0 |
|  | "72300008" | Abdominal rigidity | 52 |
|  | "438774001" | Abdominal rigidity of epigastrium | 0 |
|  | "439593009" | Abdominal rigidity of left lower quadrant | 0 |
|  | "439582007" | Abdominal rigidity of left upper quadrant | 0 |
|  | "438512007" | Abdominal rigidity of periumbilical region | 0 |
|  | "438509009" | Abdominal rigidity of right lower quadrant | 0 |
|  | "449092006" | Abdominal rigidity of right upper quadrant | 0 |
|  | "43478001" | Abdominal tenderness | 7414 |
|  | "162042000" | Abdominal wall pain | 10851 |
|  | "45979003" | Abdominal wind pain | 454 |
|  | "9209005" | Acute abdomen | 4332 |
|  | "116290004" | Acute abdominal pain | 2 |
|  | "444746004" | Acute exacerbation of chronic abdominal pain | 0 |
|  | "778641000000100" | Adnexal tenderness | 0 |
|  | "297123002" | Adnexal tenderness | 261 |
|  | "136571000119109" | Adult colic | 0 |
|  | "196791007" | Appendicular colic | 130 |
|  | "37389005" | Biliary colic | 51979 |
|  | "125661000119107" | Biliary colic with obstruction | 0 |
|  | "300459000" | Bladder tender | 4 |
|  | "25451005" | Board-like abdominal rigidity | 1 |
|  | "21005005" | Burning epigastric pain | 0 |
|  | "272047006" | C/O - loin pain | 108129 |
|  | "274278000" | C/O left iliac fossa pain | 271 |
|  | "274277005" | C/O right iliac fossa pain | 255 |
|  | "162046002" | Central abdominal pain | 31361 |
|  | "111985007" | Chronic abdominal pain | 16 |
|  | "235841007" | Chronic nonspecific abdominal pain | 615 |
|  | "958281000006114" | Colicky abdominal pain control | 33 |
|  | "958291000006112" | Colicky abdominal pain present | 479 |
|  | "371094000" | Colicky hypogastric pain | 0 |
|  | "707597009" | Continuous abdominal pain of left lower quadrant | 0 |
|  | "79922009" | Epigastric pain | 497614 |
|  | "247361008" | Evening colic | 99 |
|  | "247355005" | Flank pain | 20420 |
|  | "300348008" | Gallbladder tender | 0 |
|  | "247351001" | Generalised abdominal pain | 0 |
|  | "102614006" | Generalised abdominal pain | 24841 |
|  | "139326009" | Generalised abdominal pain | 0 |
|  | "440122009" | Generalised abdominal rigidity | 0 |
|  | "440220007" | Generalised abdominal tenderness | 0 |
|  | "371102005" | Generalised colicky abdominal pain | 0 |
|  | "271853005" | Hypochondrial pain | 22555 |
|  | "247354009" | Iliac fossa pain | 3698 |
|  | "300446008" | Kidney tender | 7 |
|  | "162049009" | Left flank pain | 21037 |
|  | "301368006" | Left hypochondrial pain | 106 |
|  | "162052001" | Left iliac fossa pain | 81166 |
|  | "301716002" | Left lower quadrant pain | 939 |
|  | "285387005" | Left sided abdominal pain | 1661 |
|  | "162047006" | Left subcostal pain | 2876 |
|  | "301715003" | Left upper quadrant pain | 5426 |
|  | "102627005" | Liver tender | 1 |
|  | "102613000" | Localised abdominal pain | 0 |
|  | "271857006" | Loin pain | 109740 |
|  | "86208007" | Loin pain-haematuria syndrome | 72 |
|  | "54586004" | Lower abdominal pain | 395226 |
|  | "60043000" | Midabdominal crampy pain | 0 |
|  | "162038003" | Non-colicky abdominal pain | 6600 |
|  | "139307001" | Non-colicky abdominal pain | 0 |
|  | "304542004" | Nonspecific abdominal pain | 34928 |
|  | "274287009" | O/E - abdominal pain | 1446 |
|  | "163248003" | O/E - abdominal rigidity | 67 |
|  | "564321000000100" | O/E - abdominal rigidity NOS | 0 |
|  | "268942007" | O/E - board-like abdominal rigidity | 470 |
|  | "274288004" | O/E - epigastric pain | 383 |
|  | "163227000" | O/E - guarding - epigastrium | 126 |
|  | "163233009" | O/E - guarding - hypogastrium | 29 |
|  | "163230007" | O/E - guarding - umbilical | 19 |
|  | "163224007" | O/E - guarding on palpation | 1654 |
|  | "275315004" | O/E - iliac pain on palpation | 149 |
|  | "163267004" | O/E - liver tender | 37 |
|  | "275316003" | O/E - lumbar pain on palpation | 670 |
|  | "164167004" | O/E - painful splenomegaly | 106 |
|  | "163239008" | O/E - rebound - epigastrium | 21 |
|  | "163245000" | O/E - rebound - hypogastrium | 15 |
|  | "163242002" | O/E - rebound - umbilical | 3 |
|  | "163236001" | O/E - rebound tenderness | 1626 |
|  | "612951000000108" | O/E - rebound tenderness NOS | 0 |
|  | "274292006" | O/E left iliac fossa tender | 221 |
|  | "163215003" | On examination - abdominal pain - epigastrium | 7272 |
|  | "163221004" | On examination - abdominal pain - hypogastrium | 1255 |
|  | "163216002" | On examination - abdominal pain - left hypochondrium | 995 |
|  | "163222006" | On examination - abdominal pain - left iliac | 3493 |
|  | "163219009" | On examination - abdominal pain - left lumbar | 375 |
|  | "163214004" | On examination - abdominal pain - right hypochondrium | 4019 |
|  | "163220003" | On examination - abdominal pain - right iliac | 4394 |
|  | "163217006" | On examination - abdominal pain - right lumbar | 427 |
|  | "163218001" | On examination - abdominal pain - umbilical | 1167 |
|  | "268941000" | On examination - abdominal pain on palpation | 34633 |
|  | "540501000000100" | On examination - abdominal pain on palpation NOS | 0 |
|  | "308903002" | On examination - epigastric pain on palpation | 6384 |
|  | "163228005" | On examination - guarding - left hypochondrium | 16 |
|  | "163234003" | On examination - guarding - left iliac | 127 |
|  | "163231006" | On examination - guarding - left lumbar | 15 |
|  | "163226009" | On examination - guarding - right hypochondrium | 95 |
|  | "163232004" | On examination - guarding - right iliac | 391 |
|  | "163229002" | On examination - guarding - right lumbar | 11 |
|  | "572781000000101" | On examination - guarding on palpation NOS | 0 |
|  | "274289007" | On examination - iliac pain - abdominal | 16 |
|  | "274290003" | On examination - lumbar pain abdominal | 7 |
|  | "163240005" | On examination - rebound-left hypochondrium | 12 |
|  | "163246004" | On examination - rebound - left iliac | 115 |
|  | "163243007" | On examination - rebound - left lumbar | 1 |
|  | "163238000" | On examination - rebound - right hypochondrium | 29 |
|  | "163244001" | On examination - rebound - right iliac | 315 |
|  | "163241009" | On examination - rebound - right lumbar | 3 |
|  | "274291004" | On examination - umbilical pain-abd. | 0 |
|  | "271859009" | Other specified abdominal pain | 0 |
|  | "695831000000102" | Other specified abdominal pain | 0 |
|  | "289842007" | Ovary tender | 0 |
|  | "838411007" | Pain in abdominal region on palpation | 0 |
|  | "838410008" | Pain in epigastric region on palpation | 0 |
|  | "667301000000109" | Pain localised to other parts of lower abdomen | 0 |
|  | "274670001" | Pain localised to other parts of lower abdomen | 0 |
|  | "247362001" | Pain on abdominal wall movement | 25 |
|  | "239231000000106" | Pain radiating to left flank | 0 |
|  | "426466001" | Pain radiating to left flank | 0 |
|  | "425860006" | Pain radiating to lower abdomen | 8 |
|  | "239271000000108" | Pain radiating to lower abdomen | 0 |
|  | "239291000000107" | Pain radiating to middle abdomen | 0 |
|  | "427668002" | Pain radiating to middle abdomen | 1 |
|  | "425834005" | Pain radiating to right flank | 0 |
|  | "239321000000102" | Pain radiating to right flank | 0 |
|  | "239381000000101" | Pain radiating to upper abdomen | 0 |
|  | "427075000" | Pain radiating to upper abdomen | 1 |
|  | "162045003" | Pain: [GIT site] or [abdominal] or [flank] or [subcostal] or [iliac fossa] | 22 |
|  | "139316002" | Pain: [site of GIT] or [abdominal site symptom] or [flank] or [subcostal] or [iliac fossa] | 0 |
|  | "296871000000108" | Percussion tenderness of abdomen | 0 |
|  | "443503005" | Periumbilical pain | 0 |
|  | "35611005" | Rebound tenderness | 12 |
|  | "301415004" | Rebound tenderness of central region | 0 |
|  | "301412001" | Rebound tenderness of epigastrium | 0 |
|  | "301418002" | Rebound tenderness of hypogastrium | 0 |
|  | "301414000" | Rebound tenderness of left hypochondrium | 0 |
|  | "301420004" | Rebound tenderness of left iliac fossa | 3 |
|  | "301417007" | Rebound tenderness of left lumbar | 0 |
|  | "301413006" | Rebound tenderness of right hypochondrium | 1 |
|  | "301419005" | Rebound tenderness of right iliac fossa | 4 |
|  | "301416003" | Rebound tenderness of right lumbar | 0 |
|  | "833290005" | Rebound tenderness of umbilical region | 0 |
|  | "439469002" | Recurrent abdominal pain | 3 |
|  | "271858001" | Recurrent acute abdominal pain | 6481 |
|  | "38654001" | Recurrent biliary colic | 0 |
|  | "162050009" | Right flank pain | 23491 |
|  | "301367001" | Right hypochondrial pain | 466 |
|  | "162051008" | Right iliac fossa pain | 109831 |
|  | "301754002" | Right lower quadrant pain | 1226 |
|  | "285388000" | Right sided abdominal pain | 1961 |
|  | "162048001" | Right subcostal pain | 8390 |
|  | "301717006" | Right upper quadrant pain | 38707 |
|  | "162039006" | Shoulder pain from abdomen | 197 |
|  | "139308006" | Shoulder pain from abdomen | 0 |
|  | "247353003" | Site of abdominal pain | 6589 |
|  | "300566002" | Spleen tender | 0 |
|  | "271852000" | Stomach cramps | 0 |
|  | "51197009" | Stomach cramps | 33707 |
|  | "247352008" | Subcostal pain | 1166 |
|  | "162053006" | Suprapubic pain | 74692 |
|  | "301406006" | Tenderness of central region | 24 |
|  | "301403003" | Tenderness of epigastrium | 8649 |
|  | "301409004" | Tenderness of hypogastrium | 232 |
|  | "301405005" | Tenderness of left hypochondrium | 21 |
|  | "301411008" | Tenderness of left iliac fossa | 110 |
|  | "439774009" | Tenderness of left lower quadrant of abdomen | 0 |
|  | "448660000" | Tenderness of left upper quadrant of abdomen | 0 |
|  | "445386003" | Tenderness of periumbilical region | 0 |
|  | "301404009" | Tenderness of right hypochondrium | 54 |
|  | "301410009" | Tenderness of right iliac fossa | 155 |
|  | "448661001" | Tenderness of right lower quadrant of abdomen | 0 |
|  | "448265002" | Tenderness of right upper quadrant of abdomen | 0 |
|  | "102615007" | Ulcer-type pain | 0 |
|  | "88522004" | Umbilical pain | 4516 |
|  | "83132003" | Upper abdominal pain | 117030 |
|  | "438506002" | Visceral abdominal pain | 0 |
| Back pain | "156651006" | (Backache NOS) or (back pain [& low]) | 0 |
|  | "268083007" | (Backache NOS) or (back pain [& low]) | 0 |
|  | "202800008" | (Backache unspecified) or (back pain unspecified & [acute]) | 0 |
|  | "247366003" | Acute back pain with sciatica | 126663 |
|  | "278862001" | Acute low back pain | 90344 |
|  | "279035001" | Acute thoracic back pain | 96527 |
|  | "139146005" | Back pain | 0 |
|  | "139148006" | Back pain | 0 |
|  | "16986008" | Back pain | 0 |
|  | "419258005" | Back pain | 0 |
|  | "399079008" | Back pain | 0 |
|  | "373644009" | Back pain - finding | 0 |
|  | "564151000000108" | Back pain without radiation NOS | 0 |
|  | "139152006" | Back pain without radiation NOS | 0 |
|  | "161895001" | Back pain without radiation NOS | 0 |
|  | "161893008" | Back pain worse on sneezing | 593 |
|  | "161891005" | Backache | 1764682 |
|  | "373634007" | Backache - disorder | 0 |
|  | "373589003" | Backache - finding | 0 |
|  | "365882001" | Backache - finding | 0 |
|  | "161889002" | Backache symptom | 0 |
|  | "536851000000100" | Backache symptom NOS | 0 |
|  | "161892003" | Backache with radiation | 21037 |
|  | "1088111000000102" | Backache without recent injury | 1 |
|  | "643261000000101" | Backache, unspecified | 0 |
|  | "267984001" | Backache, unspecified | 0 |
|  | "161894002" | C/O - low back pain | 635955 |
|  | "272048001" | C/O - lumbar pain | 0 |
|  | "161896000" | C/O - upper back ache | 22344 |
|  | "425991004" | Cervicothoracic segmental dysfunction | 0 |
|  | "134407002" | Chronic back pain | 6147 |
|  | "631000119102" | Chronic back pain greater than three months duration | 0 |
|  | "278860009" | Chronic low back pain | 149513 |
|  | "144951000000106" | Chronic mechanical low back pain | 1 |
|  | "129511000119105" | Chronic pain in coccyx for more than three months | 0 |
|  | "129501000119107" | Chronic sacral pain for greater than three months | 0 |
|  | "782661001" | Chronic sacroiliac joint pain | 0 |
|  | "1094851000000101" | Chronic sacroiliac joint pain | 1 |
|  | "136791000119103" | Chronic thoracic back pain | 0 |
|  | "418958006" | Complaining of backache | 0 |
|  | "22913005" | Dorsalgia | 0 |
|  | "135860001" | Exacerbation of backache | 2337 |
|  | "29930001000004103" | Intractable low back pain | 1 |
|  | "298311000000108" | Left sided thoracic back pain | 1 |
|  | "279039007" | Low back pain | 3167369 |
|  | "15968741000119100" | Low back pain co-occurrent and due to bilateral sciatica | 0 |
|  | "16839401000119104" | Low back pain co-occurrent with neuralgia of left sciatic nerve | 0 |
|  | "46960006" | Lumbago-sciatica due to displacement of lumbar intervertebral disc | 0 |
|  | "202794004" | Lumbago with sciatica | 190113 |
|  | "267067009" | Lumbar ache - renal | 31203 |
|  | "279063004" | Lumbar facet joint pain | 253 |
|  | "279042001" | Lumbar segmental dysfunction | 30 |
|  | "298674008" | Lumbar spine painful on movement | 33 |
|  | "103016004" | Lumbosacral nerve root pain | 0 |
|  | "279040009" | Mechanical low back pain | 152134 |
|  | "1573621000006114" | Non-mechanical back pain | 37 |
|  | "275316003" | O/E - lumbar pain on palpation | 670 |
|  | "163212000" | O/E: pain on palpation (& [abdominal] or [epigastric] or [iliac] or [lumbar] or [umbilical]) | 153 |
|  | "830292003" | Pain in left lumbar region | 0 |
|  | "838412000" | Pain in lumbar region on palpation | 0 |
|  | "267982002" | Pain in lumbar spine | 217501 |
|  | "830293008" | Pain in right lumbar region | 0 |
|  | "34789001" | Pain in the coccyx | 93234 |
|  | "267981009" | Pain in thoracic spine | 57688 |
|  | "427475007" | Pain radiating to lumbar region of back | 0 |
|  | "239281000000105" | Pain radiating to lumbar region of back | 0 |
|  | "247368002" | Posterior compartment low back pain | 1 |
|  | "300957005" | Postural low back pain | 246 |
|  | "298301000000106" | Right sided thoracic back pain | 0 |
|  | "61486003" | Sacral back pain | 441 |
|  | "3200003" | Sacrocoxalgia | 0 |
|  | "202487003" | Sacroiliac joint pain | 11736 |
|  | "279038004" | Thoracic back pain | 18087 |
|  | "298254008" | Thoracic facet joint pain | 71 |
|  | "279037009" | Thoracic segmental dysfunction | 14 |
|  | "298579007" | Thoracic spine - painful on movement | 40 |
| Bone pain | "202793005" | (Back pain: [lumbar spine] or [low] or [acute lumbar]) or (lumbalgia) or (lumbago) | 2810 |
|  | "15743681000119106" | Bilateral pain of shoulder blades | 0 |
|  | "12584003" | Bone pain | 6794 |
|  | "278997003" | Bone tenderness | 55 |
|  | "203508001" | Bony pelvic pain | 1729 |
|  | "298477006" | Cervical spine tender | 137 |
|  | "102554000" | Cervical spinous process tenderness | 0 |
|  | "129511000119105" | Chronic pain in coccyx for more than three months | 0 |
|  | "203509009" | Clavicle pain | 5665 |
|  | "161977000" | Costal margin chest pain | 6807 |
|  | "22913005" | Dorsalgia | 0 |
|  | "282743009" | Malignant bone pain | 31 |
|  | "279029001" | Pain in cervical spine | 12781 |
|  | "301773003" | Pain in femur | 19 |
|  | "267982002" | Pain in lumbar spine | 217501 |
|  | "202764005" | Pain in neck (& [cervical spine]) | 17792 |
|  | "48926009" | Pain in spine | 273 |
|  | "34789001" | Pain in the coccyx | 93234 |
|  | "156647009" | Pain in thoracic spine | 0 |
|  | "267981009" | Pain in thoracic spine | 57688 |
|  | "774135008" | Pain of left shoulder blade | 0 |
|  | "774134007" | Pain of right shoulder blade | 0 |
|  | "298731003" | Pain of sternum | 183 |
|  | "710230000" | Painful os peroneum syndrome | 0 |
|  | "297217002" | Rib pain | 159886 |
|  | "3200003" | Sacrocoxalgia | 0 |
|  | "20793008" | Scapulalgia | 9 |
|  | "202792000" | Thoracic pain: [spine] or [acute back] | 1521 |
|  | "298578004" | Thoracic spine - tender | 140 |
|  | "89638008" | Xiphodynia | 0 |
|  | "89874002" | Xiphoidalgia syndrome | 14 |
| Abdominal pain (upper) | "307724003" | [D] Left upper quadrant pain | 0 |
|  | "307722004" | [D] Right upper quadrant pain | 0 |
|  | "307725002" | [D] Upper abdominal pain | 0 |
|  | "207211000" | [D]Epigastric pain | 0 |
|  | "499161000000104" | [D]Epigastric pain | 0 |
|  | "499181000000108" | [D]Hypochondrial pain | 0 |
|  | "207213002" | [D]Hypochondrial pain | 0 |
|  | "502581000000102" | [D]Left upper quadrant pain | 0 |
|  | "502571000000104" | [D]Right upper quadrant pain | 1 |
|  | "502591000000100" | [D]Upper abdominal pain | 1 |
|  | "21005005" | Burning epigastric pain | 0 |
|  | "79922009" | Epigastric pain | 497614 |
|  | "271853005" | Hypochondrial pain | 22555 |
|  | "301368006" | Left hypochondrial pain | 106 |
|  | "162047006" | Left subcostal pain | 2876 |
|  | "301715003" | Left upper quadrant pain | 5426 |
|  | "274288004" | O/E - epigastric pain | 383 |
|  | "163227000" | O/E - guarding - epigastrium | 126 |
|  | "140437001" | O/E - guarding - epigastrium | 0 |
|  | "140422000" | O/E pain: [abdominal (& on palpation)] or [epigastric] or [iliac] or [lumbar] or [umbilical] | 0 |
|  | "269018004" | O/E pain: [abdominal (& on palpation)] or [epigastric] or [iliac] or [lumbar] or [umbilical] | 0 |
|  | "163215003" | On examination - abdominal pain - epigastrium | 7272 |
|  | "163221004" | On examination - abdominal pain - hypogastrium | 1255 |
|  | "163216002" | On examination - abdominal pain - left hypochondrium | 995 |
|  | "163214004" | On examination - abdominal pain - right hypochondrium | 4019 |
|  | "308903002" | On examination - epigastric pain on palpation | 6384 |
|  | "838410008" | Pain in epigastric region on palpation | 0 |
|  | "301367001" | Right hypochondrial pain | 466 |
|  | "162048001" | Right subcostal pain | 8390 |
|  | "301717006" | Right upper quadrant pain | 38707 |
|  | "247352008" | Subcostal pain | 1166 |
|  | "102615007" | Ulcer-type pain | 0 |
|  | "83132003" | Upper abdominal pain | 117030 |
| Postmenopausal bleeding | "76742009" | Postmenopausal bleeding | 175918 |
|  | "176471000000103" | Postmenopausal postcoital bleeding | 0 |
|  | "415149004" | Postmenopausal postcoital bleeding | 242 |
| Retention | "207167004" | [D]Acute retention of urine | 0 |
|  | "498821000000107" | [D]Acute retention of urine | 9 |
|  | "498831000000109" | [D]Chronic retention of urine | 0 |
|  | "207168009" | [D]Chronic retention of urine | 0 |
|  | "207164006" | [D]Clot retention of urine | 0 |
|  | "498801000000103" | [D]Clot retention of urine | 0 |
|  | "369111000000107" | [D]Fowler's syndrome | 0 |
|  | "339591000000102" | [D]Fowler's Syndrome | 0 |
|  | "498791000000102" | [D]Retention of urine | 0 |
|  | "207163000" | [D]Retention of urine | 0 |
|  | "207169001" | [D]Retention of urine unspecified | 0 |
|  | "498841000000100" | [D]Retention of urine unspecified | 0 |
|  | "236649000" | Acute-on-chronic retention of urine | 373 |
|  | "236648008" | Acute retention of urine | 31396 |
|  | "130951007" | Bladder retention of urine | 7 |
|  | "66748007" | Bladder retention of urine | 0 |
|  | "782390002" | Cauda equina syndrome with painless urinary retention | 0 |
|  | "1093161000000109" | Cauda equina syndrome with painless urinary retention | 0 |
|  | "236650000" | Chronic retention of urine | 9274 |
|  | "236651001" | Clot retention of urine | 1079 |
|  | "885951000000107" | Fowler's syndrome | 0 |
|  | "700242002" | Fowler syndrome | 547 |
|  | "267064002" | Retention of urine | 79485 |
|  | "367421000119109" | Retention of urine caused by drug | 0 |
|  | "386490009" | Urinary retention care | 0 |
|  | "762226007" | Urinary retention care management | 0 |
|  | "796511000000109" | Urinary retention care management | 0 |
|  | "117551000119108" | Urinary retention due to benign prostatic hypertrophy | 1 |
| Shortness of breath | "502631000000100" | [D]Breathlessness | 0 |
|  | "308149007" | [D]Breathlessness | 0 |
|  | "207059009" | [D]Dyspnoea | 0 |
|  | "498021000000103" | [D]Dyspnoea | 0 |
|  | "497941000000108" | [D]Orthopnoea | 0 |
|  | "207051007" | [D]Orthopnoea | 0 |
|  | "207057006" | [D]Shortness of breath | 0 |
|  | "498001000000107" | [D]Shortness of breath | 0 |
|  | "71646001" | AIDS with dyspnoea | 0 |
|  | "887741000000102" | Anxiety about breathlessness | 0 |
|  | "887751000000104" | Anxiety about breathlessness | 0 |
|  | "702535006" | Anxiety about breathlessness | 165 |
|  | "401323002" | Borg Breathlessness Score: 0.5 very, very slight (just noticeable) | 510 |
|  | "401279002" | Borg Breathlessness Score: 1 very slight | 643 |
|  | "401293009" | Borg Breathlessness Score: 10 maximal | 15 |
|  | "401280004" | Borg Breathlessness Score: 2 slight | 1167 |
|  | "401281000" | Borg Breathlessness Score: 3 moderate | 2678 |
|  | "401282007" | Borg Breathlessness Score: 4 somewhat severe | 676 |
|  | "401284008" | Borg Breathlessness Score: 5 severe | 391 |
|  | "401286005" | Borg Breathlessness Score: 6 severe (+) | 98 |
|  | "401290007" | Borg Breathlessness Score: 7 very severe | 183 |
|  | "401291006" | Borg Breathlessness Score: 8 very severe (+) | 53 |
|  | "401292004" | Borg Breathlessness Score: 9 very, very severe (almost maximal) | 25 |
|  | "161940008" | Breathless - mild exertion | 122743 |
|  | "161939006" | Breathless - moderate exertion | 183272 |
|  | "390871002" | Breathless - strenuous exertion | 31232 |
|  | "719413006" | Breathlessness care management | 0 |
|  | "1047261000000103" | Breathlessness care management | 0 |
|  | "1861181000006114" | Breathlessness causing anxiety | 22 |
|  | "1861171000006111" | Breathlessness causing difficulty eating | 0 |
|  | "888131000000106" | Breathlessness causing difficulty eating | 0 |
|  | "888141000000102" | Breathlessness causing difficulty eating | 0 |
|  | "572661000000100" | Breathlessness NOS | 0 |
|  | "248584002" | Catching breath | 41 |
|  | "870535009" | Chronic dyspnoea | 0 |
|  | "230145002" | Difficulty breathing | 90649 |
|  | "161945003" | Difficulty breathing | 0 |
|  | "139200001" | Difficulty breathing | 0 |
|  | "702581006" | Difficulty eating due to breathlessness | 13 |
|  | "49233005" | Dyspnoea | 0 |
|  | "267036007" | Dyspnoea | 1114900 |
|  | "57769004" | Dyspnoea after eating | 0 |
|  | "422177004" | Dyspnoea associated with AIDS | 0 |
|  | "161941007" | Dyspnoea at rest | 16426 |
|  | "119981000146107" | Dyspnoea caused by SARS-CoV-2 (severe acute respiratory syndrome coronavirus 2) | 0 |
|  | "20112008" | Dyspnoea leaning over | 0 |
|  | "60845006" | Dyspnoea on exertion | 171737 |
|  | "24921003" | Dyspnoea raising arms | 0 |
|  | "72365000" | Dyspnoea, class II | 0 |
|  | "39950000" | Dyspnoea, class III | 0 |
|  | "73322006" | Dyspnoea, class IV | 0 |
|  | "1089971000000104" | eMRC (extended Medical Research Council) dyspnoea scale grade 1 | 0 |
|  | "1089981000000102" | eMRC (extended Medical Research Council) dyspnoea scale grade 2 | 0 |
|  | "1089991000000100" | eMRC (extended Medical Research Council) dyspnoea scale grade 3 | 0 |
|  | "1090011000000104" | eMRC (extended Medical Research Council) dyspnoea scale grade 4 | 0 |
|  | "1090001000000101" | eMRC (extended Medical Research Council) dyspnoea scale grade 5a | 0 |
|  | "1090021000000105" | eMRC (extended Medical Research Council) dyspnoea scale grade 5b | 0 |
|  | "34560001" | Expiratory dyspnoea | 0 |
|  | "23141003" | Gasping for breath | 119 |
|  | "297216006" | Increasing breathlessness | 1592 |
|  | "25209001" | Inspiratory dyspnoea | 0 |
|  | "391120009" | Medical Research Council Dyspnoea scale grade 1 | 285361 |
|  | "391123006" | Medical Research Council Dyspnoea scale grade 2 | 478976 |
|  | "391124000" | Medical Research Council Dyspnoea scale grade 3 | 317997 |
|  | "391125004" | Medical Research Council Dyspnoea scale grade 4 | 160388 |
|  | "391126003" | Medical Research Council Dyspnoea scale grade 5 | 34791 |
|  | "852051000000107" | Minimal breathlessness | 0 |
|  | "1099381000000100" | mMRC (modified Medical Research Council) dyspnoea scale grade 1 | 11 |
|  | "1099391000000103" | mMRC (modified Medical Research Council) dyspnoea scale grade 2 | 18 |
|  | "1099401000000100" | mMRC (modified Medical Research Council) dyspnoea scale grade 3 | 21 |
|  | "1099411000000103" | mMRC (modified Medical Research Council) dyspnoea scale grade 4 | 12 |
|  | "1060991000000107" | MRC (Medical Research Council) Breathlessness Scale grade 5a | 3 |
|  | "1061001000000106" | MRC (Medical Research Council) Breathlessness Scale grade 5b | 0 |
|  | "248548009" | Nocturnal dyspnoea | 3994 |
|  | "162890008" | O/E - dyspnoea | 5043 |
|  | "1807841000006119" | O/E - dyspnoea at rest | 28 |
|  | "1807851000006117" | O/E - dyspnoea on exertion | 76 |
|  | "162891007" | O/E - orthopnoea | 810 |
|  | "1049241000000109" | Opioid therapy for breathlessness management | 0 |
|  | "719416003" | Opioid therapy for breathlessness management | 0 |
|  | "62744007" | Orthopnoea | 11103 |
|  | "59265000" | Paroxysmal dyspnoea | 0 |
|  | "55442000" | Paroxysmal nocturnal dyspnoea | 5916 |
|  | "30744009" | Platypnoea | 0 |
|  | "390870001" | Short of breath dressing/undressing | 1304 |
|  | "102577000" | Trepopnoea | 0 |
|  | "407588003" | Unable to complete a sentence in one breath | 953 |
| Testicular mass | "207202000" | [D]Swelling of scrotum | 0 |
|  | "499091000000106" | [D]Swelling of scrotum | 0 |
|  | "773611000000105" | C/O scrotal swelling | 4541 |
|  | "773621000000104" | C/O scrotal swelling | 0 |
|  | "366563009" | Inflammatory testicular mass | 0 |
|  | "309533009" | Mass of testis | 0 |
|  | "390919006" | O/E - left scrotal swelling | 592 |
|  | "390918003" | O/E - right scrotal swelling | 485 |
|  | "268946005" | O/E - scrotal swelling | 21550 |
|  | "589841000000103" | O/E - scrotal swelling NOS | 0 |
|  | "163378005" | O/E - testicular swelling | 9262 |
|  | "140587006" | O/E - testicular swelling | 0 |
|  | "140586002" | O/E -scrotal swell.no transill | 0 |
|  | "163376009" | O/E -scrotal swelling-transill | 303 |
|  | "163374007" | O/E swelling: [scrotal] or [epididymal] or [testicular] | 25 |
|  | "140583005" | O/E swelling: [scrotal] or [epididymal] or [testicular] | 0 |
|  | "163377000" | On examination - scrotal swelling - no transillumination | 152 |
|  | "197984006" | Orchitis | 0 |
|  | "102031000119109" | Paratesticular mass | 0 |
|  | "53929009" | Scrotal mass | 826 |
|  | "271687003" | Swelling of scrotum | 7766 |
|  | "706671000000100" | Swelling of scrotum | 0 |
|  | "438457000" | Swelling of testicle | 34046 |
|  | "163384008" | Testicular lump | 0 |
|  | "140593003" | Testicular lump | 0 |
|  | "87860000" | Testicular mass | 76708 |
|  | "276377005" | Testicular mass | 0 |
|  | "274756006" | Testicular swelling | 0 |
| Testicular pain | "722829006" | Acute pain of scrotum | 1 |
|  | "16675251000119106" | Bilateral testicular pain | 0 |
|  | "20502007" | Pain in scrotum | 16233 |
|  | "139430006" | Pain in scrotum | 0 |
|  | "162150007" | Pain in scrotum | 0 |
|  | "63901009" | Pain in testicle | 209116 |
|  | "16675301000119100" | Pain of left testicle | 0 |
|  | "16675201000119107" | Pain of right testicle | 0 |
|  | "282360001" | Persistent testicular pain | 593 |
|  | "247396008" | Testicular neuralgia | 16 |
| Urinary frequency | "207176006" | [D]Frequency of micturition, unspecified | 0 |
|  | "498891000000105" | [D]Frequency of micturition, unspecified | 0 |
|  | "162116003" | Increased frequency of urination | 298330 |
|  | "117531000119102" | Urinary frequency due to benign prostatic hypertrophy | 0 |
| Urinary urgency | "207173003" | [D] Urge incontinence | 0 |
|  | "498871000000106" | [D]Urge incontinence | 0 |
|  | "499001000000104" | [D]Urgency of micturition | 0 |
|  | "207189000" | [D]Urgency of micturition | 0 |
|  | "236659004" | Cough - urge incontinence of urine | 81 |
|  | "762265005" | Mixed incontinence due to prolapse of female genital organ | 0 |
|  | "413343005" | Mixed urinary incontinence | 3790 |
|  | "762264009" | Urge incontinence due to prolapse of female genital organ | 0 |
|  | "87557004" | Urge incontinence of urine | 80162 |
|  | "299271000000100" | Urge to pass urine again shortly after finishing voiding | 1502 |
|  | "299281000000103" | Urge to pass urine again shortly after finishing voiding | 0 |
|  | "299291000000101" | Urge to pass urine again shortly after finishing voiding | 0 |
|  | "75088002" | Urgent desire to urinate | 190024 |
|  | "117511000119107" | Urinary urgency due to benign prostatic hypertrophy | 0 |
| Weight loss | "158271000" | [D]Abnormal loss of weight | 0 |
|  | "206919000" | [D]Abnormal loss of weight | 0 |
|  | "496901000000107" | [D]Abnormal loss of weight | 0 |
|  | "23712001" | Abnormal decrease in weight | 0 |
|  | "267024001" | Abnormal weight loss | 216541 |
|  | "161834000" | Abnormal weight loss (& [symptom]) | 0 |
|  | "198511000000103" | Complaining of weight loss | 27198 |
|  | "213791000000109" | Complaining of weight loss | 0 |
|  | "213801000000108" | Complaining of weight loss | 0 |
|  | "699205002" | Involuntary weight loss | 0 |
|  | "422868009" | Unexplained weight loss | 2057 |
|  | "511461000000103" | Unexplained weight loss | 0 |
|  | "768571000000103" | Unintentional weight loss | 0 |
|  | "768581000000101" | Unintentional weight loss | 0 |
|  | "448765001" | Unintentional weight loss | 13841 |
|  | "161832001" | Weight decreasing | 60167 |
|  | "139089007" | Weight decreasing | 0 |
|  | "139091004" | Weight loss (& abnormal) | 0 |
|  | "267158006" | Weight loss (& abnormal) | 0 |
| Fatigue | "495771000000107" | [D]Fatigue | 1 |
|  | "206767001" | [D]Fatigue | 0 |
|  | "206765009" | [D]Malaise and fatigue | 0 |
|  | "158167007" | [D]Malaise and fatigue | 0 |
|  | "495751000000103" | [D]Malaise and fatigue | 0 |
|  | "414631000000100" | [D]Malaise and fatigue NOS | 0 |
|  | "463761000000103" | [D]Malaise and fatigue NOS | 1 |
|  | "158173008" | [D]Malaise and fatigue NOS | 0 |
|  | "206773000" | [D]Malaise and fatigue NOS | 0 |
|  | "206770002" | [D]Postviral (asthenic) syndrome | 0 |
|  | "158172003" | [D]Postviral (asthenic) syndrome | 0 |
|  | "495791000000106" | [D]Postviral (asthenic) syndrome | 2 |
|  | "495801000000105" | [D]Tiredness | 4 |
|  | "206771003" | [D]Tiredness | 0 |
|  | "73266005" | AIDS with fatigue | 0 |
|  | "272062008" | C/O - "tired all the time" | 79686 |
|  | "716749005" | Cancer-related fatigue | 0 |
|  | "52702003" | Chronic fatigue syndrome | 129752 |
|  | "84229001" | Fatigue | 223391 |
|  | "272060000" | Fatigue - symptom | 45192 |
|  | "420900006" | Fatigue associated with AIDS | 0 |
|  | "784317004" | Fatigue due to chemotherapy | 0 |
|  | "97201000119101" | Fatigue due to chemotherapy or radiation therapy | 0 |
|  | "784318009" | Fatigue due to radiation therapy | 0 |
|  | "704369007" | Fatigue due to treatment | 0 |
|  | "88895004" | Fatigue during pregnancy | 332 |
|  | "199121007" | Fatigue during pregnancy - delivered | 0 |
|  | "199122000" | Fatigue during pregnancy - delivered with postnatal complication | 0 |
|  | "199123005" | Fatigue during pregnancy - not delivered | 9 |
|  | "698941000000107" | Fatigue during pregnancy unspecified | 0 |
|  | "199124004" | Fatigue during pregnancy with postnatal complication | 1 |
|  | "314109004" | Feeling tired | 2916 |
|  | "271795006" | Malaise and fatigue | 88829 |
|  | "491281000000101" | Mild chronic fatigue syndrome | 0 |
|  | "377181000000104" | Mild chronic fatigue syndrome | 648 |
|  | "377171000000101" | Moderate chronic fatigue syndrome | 450 |
|  | "491291000000104" | Moderate chronic fatigue syndrome | 0 |
|  | "713568000" | Occasionally tired | 0 |
|  | "508581000000104" | Post-exertional fatigue | 0 |
|  | "444042007" | Postexertional fatigue | 0 |
|  | "51771007" | Postviral fatigue syndrome | 33067 |
|  | "442099003" | Psychogenic fatigue | 0 |
|  | "480621000000108" | Severe chronic fatigue syndrome | 0 |
|  | "377161000000108" | Severe chronic fatigue syndrome | 159 |
|  | "224960004" | Tired | 3160 |
|  | "267032009" | Tired all the time | 810720 |
|  | "248269005" | Tired on least exertion | 231 |
|  | "267031002" | Tiredness symptom | 578442 |
|  | "580521000000101" | Tiredness symptom NOS | 0 |
| Hoarseness | "206974000" | [D]Hoarseness | 0 |
|  | "497331000000103" | [D]Hoarseness | 1 |
|  | "1371000119105" | Chronic hoarseness | 0 |
|  | "50219008" | Hoarse | 177348 |
|  | "542871000000109" | Hoarseness symptom NOS | 0 |
|  | "59176006" | Low-pitch hoarse group | 0 |
|  | "164276001" | O/E - hoarseness | 4050 |
|  | "57568005" | Strained hoarse voice-continuous group | 0 |
|  | "68391009" | Strained hoarse voice arrest-intermittent arrhythmic group | 0 |
|  | "64184006" | Strained hoarse voice arrest-intermittent rhythmic group | 0 |
| Iron deficiency anaemia | "191408005" | [X]Other iron deficiency anaemias | 0 |
|  | "397761000000103" | [X]Other iron deficiency anaemias | 0 |
|  | "724557008" | Acquired iron deficiency anaemia due to decreased absorption | 0 |
|  | "717948004" | Acquired iron deficiency anaemia due to increased iron requirement | 0 |
|  | "413533008" | Anaemia due to chronic blood loss | 8565 |
|  | "191127009" | Anaemia due to chronic blood loss: [iron deficiency] or [normocytic] | 114 |
|  | "44252001" | Blood loss anaemia | 0 |
|  | "191135007" | Chlorotic anaemia | 0 |
|  | "722005000" | Iron-refractory iron deficiency anaemia | 3 |
|  | "87522002" | Iron deficiency anaemia | 597073 |
|  | "724556004" | Iron deficiency anaemia due to blood loss | 0 |
|  | "234351006" | Iron deficiency anaemia due to chronic blood loss | 0 |
|  | "191128004" | Iron deficiency anaemia due to dietary causes | 4023 |
|  | "598461000000107" | Iron deficiency anaemia NOS | 0 |
|  | "42626004" | Iron deficiency anaemia secondary to chronic blood loss | 0 |
|  | "371315009" | Iron deficiency anaemia secondary to inadequate dietary iron intake | 0 |
|  | "661301000000100" | Other specified iron deficiency anaemia | 0 |
|  | "610661000000100" | Other specified iron deficiency anaemia NOS | 0 |
|  | "80126007" | Plummer-Vinson syndrome | 154 |
|  | "610671000000107" | Unspecified iron deficiency anaemia | 0 |

**ST2: Clinical feature consultation rate ratios (CRR) comparing periods of time in 2020 with 2019.** Clinical features are sorted by descending CRR in the whole year.

| **Clinical feature** | **CRR   (weeks 1-52)  [All Year]** | **CRR   (weeks 1-12)  [Pre-lockdown]** | **CRR   (weeks 13-52)  [Post-lockdown]** | **CRR   (weeks 1-6)  [Jan/Feb]** | **CRR   (weeks 7-12)  [Feb/Mar]** | **CRR   (weeks 13-18)  [Apr/May]** | **CRR   (weeks 19-24)  [May/June]** | **CRR   (weeks 25-30)  [Jun/Jul]** | **CRR   (weeks 31-36)  [Aug/Sep]** | **CRR   (weeks 37-42)  [Sep/Oct]** | **CRR   (weeks 43-52)  [Oct-Dec]** |
| --- | --- | --- | --- | --- | --- | --- | --- | --- | --- | --- | --- |
| Constipation | 0.99 (0.98 to 1.00) | 0.88 (0.86 to 0.89) | 1.03 (1.02 to 1.04) | 0.94 (0.92 to 0.96) | 0.81 (0.79 to 0.83) | 0.82 (0.80 to 0.84) | 1.02 (0.99 to 1.05) | 1.08 (1.06 to 1.11) | 1.03 (1.00 to 1.05) | 1.02 (1.00 to 1.05) | 1.14 (1.12 to 1.16) |
| Dysphagia | 0.99 (0.97 to 1.01) | 1.06 (1.01 to 1.10) | 0.96 (0.94 to 0.99) | 1.10 (1.04 to 1.17) | 1.01 (0.95 to 1.06) | 0.68 (0.63 to 0.73) | 0.91 (0.85 to 0.97) | 1.01 (0.95 to 1.07) | 1.08 (1.01 to 1.14) | 1.06 (1.00 to 1.12) | 1.00 (0.96 to 1.05) |
| Breast lump (f) | 0.95 (0.94 to 0.97) | 0.80 (0.77 to 0.82) | 1.01 (0.99 to 1.02) | 0.89 (0.85 to 0.93) | 0.70 (0.67 to 0.74) | 0.64 (0.61 to 0.68) | 0.83 (0.80 to 0.87) | 1.04 (0.99 to 1.08) | 1.08 (1.04 to 1.13) | 1.09 (1.05 to 1.14) | 1.22 (1.18 to 1.26) |
| Jaundice | 0.95 (0.91 to 1.00) | 0.94 (0.86 to 1.04) | 0.95 (0.90 to 1.01) | 1.06 (0.92 to 1.22) | 0.84 (0.74 to 0.97) | 0.75 (0.65 to 0.87) | 1.05 (0.91 to 1.21) | 0.90 (0.78 to 1.04) | 0.91 (0.79 to 1.05) | 1.01 (0.87 to 1.16) | 1.08 (0.96 to 1.21) |
| Rectal bleeding | 0.94 (0.93 to 0.96) | 0.89 (0.87 to 0.92) | 0.96 (0.95 to 0.98) | 0.94 (0.91 to 0.98) | 0.84 (0.81 to 0.87) | 0.61 (0.59 to 0.64) | 0.85 (0.82 to 0.89) | 1.01 (0.97 to 1.05) | 1.05 (1.01 to 1.10) | 0.99 (0.95 to 1.03) | 1.13 (1.10 to 1.17) |
| Frank haematuria | 0.94 (0.90 to 0.98) | 0.94 (0.86 to 1.02) | 0.95 (0.90 to 0.99) | 0.99 (0.88 to 1.10) | 0.89 (0.79 to 1.00) | 0.73 (0.64 to 0.83) | 1.02 (0.90 to 1.15) | 1.00 (0.88 to 1.13) | 0.88 (0.77 to 0.99) | 1.02 (0.90 to 1.15) | 1.00 (0.91 to 1.10) |
| Testicular mass or pain (m) | 0.92 (0.91 to 0.94) | 0.74 (0.72 to 0.77) | 0.99 (0.97 to 1.01) | 0.78 (0.74 to 0.82) | 0.70 (0.66 to 0.74) | 0.68 (0.64 to 0.73) | 0.83 (0.78 to 0.88) | 0.95 (0.90 to 1.01) | 1.09 (1.03 to 1.16) | 1.06 (1.01 to 1.12) | 1.21 (1.16 to 1.26) |
| Nausea | 0.91 (0.89 to 0.93) | 0.89 (0.86 to 0.92) | 0.92 (0.90 to 0.94) | 0.95 (0.91 to 1.00) | 0.82 (0.78 to 0.86) | 0.72 (0.68 to 0.76) | 0.75 (0.71 to 0.79) | 0.94 (0.89 to 0.99) | 0.99 (0.94 to 1.04) | 0.98 (0.94 to 1.03) | 1.03 (0.99 to 1.07) |
| Shortness of breath | 0.89 (0.88 to 0.89) | 0.98 (0.97 to 0.99) | 0.85 (0.85 to 0.86) | 1.04 (1.02 to 1.05) | 0.92 (0.91 to 0.94) | 0.88 (0.86 to 0.89) | 0.90 (0.89 to 0.92) | 0.83 (0.82 to 0.84) | 0.82 (0.80 to 0.83) | 0.82 (0.81 to 0.84) | 0.86 (0.85 to 0.87) |
| Appetite loss | 0.87 (0.85 to 0.89) | 0.91 (0.87 to 0.95) | 0.86 (0.84 to 0.89) | 0.94 (0.89 to 1.00) | 0.87 (0.82 to 0.92) | 0.75 (0.69 to 0.81) | 0.77 (0.71 to 0.83) | 0.88 (0.82 to 0.94) | 1.03 (0.96 to 1.10) | 0.98 (0.91 to 1.05) | 0.82 (0.78 to 0.86) |
| Lower urinary tract (m) | 0.86 (0.85 to 0.88) | 0.93 (0.90 to 0.95) | 0.84 (0.83 to 0.86) | 0.95 (0.91 to 0.99) | 0.90 (0.87 to 0.94) | 0.52 (0.49 to 0.54) | 0.66 (0.63 to 0.69) | 0.89 (0.85 to 0.93) | 0.89 (0.86 to 0.93) | 0.90 (0.87 to 0.93) | 1.03 (1.00 to 1.06) |
| Abdominal pain | 0.83 (0.83 to 0.84) | 0.83 (0.83 to 0.84) | 0.83 (0.83 to 0.84) | 0.93 (0.92 to 0.94) | 0.74 (0.72 to 0.75) | 0.52 (0.51 to 0.53) | 0.73 (0.71 to 0.74) | 0.88 (0.87 to 0.90) | 0.90 (0.89 to 0.91) | 0.88 (0.87 to 0.90) | 0.98 (0.97 to 1.00) |
| Weight loss | 0.83 (0.82 to 0.85) | 0.82 (0.79 to 0.85) | 0.84 (0.82 to 0.85) | 0.94 (0.90 to 0.99) | 0.69 (0.65 to 0.73) | 0.45 (0.42 to 0.48) | 0.83 (0.79 to 0.88) | 0.91 (0.86 to 0.95) | 0.97 (0.92 to 1.02) | 0.88 (0.84 to 0.93) | 0.90 (0.86 to 0.94) |
| Change in bowel habit | 0.82 (0.81 to 0.84) | 0.95 (0.92 to 0.99) | 0.78 (0.77 to 0.80) | 1.04 (0.99 to 1.09) | 0.86 (0.82 to 0.91) | 0.39 (0.37 to 0.42) | 0.59 (0.56 to 0.63) | 0.75 (0.71 to 0.79) | 0.91 (0.87 to 0.96) | 0.91 (0.87 to 0.96) | 1.01 (0.97 to 1.05) |
| Postmenopausal bleeding (f) | 0.81 (0.79 to 0.83) | 0.97 (0.92 to 1.03) | 0.76 (0.73 to 0.78) | 1.08 (1.00 to 1.16) | 0.87 (0.81 to 0.95) | 0.54 (0.49 to 0.59) | 0.68 (0.63 to 0.74) | 0.76 (0.70 to 0.82) | 0.81 (0.74 to 0.88) | 0.80 (0.74 to 0.87) | 0.88 (0.83 to 0.94) |
| Distension (f) | 0.80 (0.78 to 0.81) | 0.75 (0.71 to 0.79) | 0.81 (0.79 to 0.83) | 0.85 (0.79 to 0.91) | 0.66 (0.61 to 0.71) | 0.35 (0.32 to 0.39) | 0.67 (0.63 to 0.72) | 0.79 (0.74 to 0.84) | 0.90 (0.84 to 0.96) | 0.90 (0.84 to 0.96) | 1.13 (1.07 to 1.20) |
| Haemoptysis | 0.80 (0.77 to 0.82) | 0.91 (0.86 to 0.97) | 0.75 (0.72 to 0.78) | 0.95 (0.87 to 1.03) | 0.87 (0.79 to 0.95) | 0.60 (0.54 to 0.67) | 0.73 (0.65 to 0.81) | 0.85 (0.76 to 0.95) | 0.83 (0.74 to 0.93) | 0.86 (0.78 to 0.95) | 0.72 (0.67 to 0.78) |
| Back pain | 0.79 (0.79 to 0.80) | 0.87 (0.86 to 0.88) | 0.77 (0.76 to 0.77) | 0.97 (0.96 to 0.99) | 0.76 (0.75 to 0.77) | 0.52 (0.51 to 0.53) | 0.66 (0.65 to 0.67) | 0.80 (0.79 to 0.81) | 0.82 (0.80 to 0.83) | 0.84 (0.83 to 0.86) | 0.90 (0.89 to 0.91) |
| Bone pain | 0.79 (0.77 to 0.80) | 0.91 (0.88 to 0.94) | 0.75 (0.73 to 0.76) | 1.00 (0.95 to 1.05) | 0.83 (0.78 to 0.87) | 0.47 (0.44 to 0.50) | 0.62 (0.59 to 0.66) | 0.75 (0.71 to 0.80) | 0.78 (0.74 to 0.83) | 0.88 (0.83 to 0.92) | 0.90 (0.86 to 0.93) |
| Indigestion | 0.78 (0.77 to 0.79) | 0.80 (0.77 to 0.82) | 0.77 (0.76 to 0.78) | 0.86 (0.82 to 0.89) | 0.73 (0.70 to 0.76) | 0.57 (0.54 to 0.60) | 0.75 (0.72 to 0.79) | 0.81 (0.78 to 0.85) | 0.81 (0.77 to 0.85) | 0.78 (0.75 to 0.82) | 0.85 (0.82 to 0.88) |
| Iron deficiency anaemia | 0.76 (0.75 to 0.78) | 0.94 (0.92 to 0.97) | 0.71 (0.70 to 0.72) | 1.00 (0.97 to 1.04) | 0.88 (0.85 to 0.92) | 0.40 (0.38 to 0.42) | 0.54 (0.52 to 0.57) | 0.69 (0.66 to 0.72) | 0.79 (0.76 to 0.83) | 0.82 (0.79 to 0.85) | 0.92 (0.89 to 0.95) |
| Abdominal pain (upper) | 0.75 (0.74 to 0.76) | 0.83 (0.81 to 0.86) | 0.72 (0.71 to 0.74) | 0.91 (0.87 to 0.96) | 0.74 (0.71 to 0.78) | 0.47 (0.44 to 0.50) | 0.65 (0.61 to 0.69) | 0.78 (0.74 to 0.82) | 0.73 (0.69 to 0.77) | 0.75 (0.71 to 0.79) | 0.85 (0.82 to 0.89) |
| Diarrhoea | 0.75 (0.74 to 0.76) | 0.85 (0.83 to 0.87) | 0.72 (0.71 to 0.73) | 0.92 (0.90 to 0.94) | 0.78 (0.76 to 0.80) | 0.56 (0.54 to 0.57) | 0.64 (0.62 to 0.66) | 0.73 (0.71 to 0.74) | 0.80 (0.78 to 0.82) | 0.76 (0.74 to 0.78) | 0.79 (0.77 to 0.81) |
| Fatigue | 0.69 (0.69 to 0.70) | 0.82 (0.81 to 0.84) | 0.65 (0.64 to 0.66) | 1.01 (0.98 to 1.03) | 0.67 (0.65 to 0.69) | 0.38 (0.37 to 0.39) | 0.48 (0.47 to 0.49) | 0.63 (0.61 to 0.64) | 0.73 (0.72 to 0.75) | 0.78 (0.76 to 0.80) | 0.87 (0.85 to 0.89) |
| Hoarse | 0.65 (0.63 to 0.67) | 0.92 (0.87 to 0.97) | 0.56 (0.54 to 0.58) | 1.06 (0.98 to 1.14) | 0.78 (0.72 to 0.85) | 0.37 (0.33 to 0.41) | 0.57 (0.52 to 0.63) | 0.62 (0.57 to 0.68) | 0.70 (0.64 to 0.76) | 0.59 (0.54 to 0.64) | 0.54 (0.50 to 0.58) |
| Cough | 0.61 (0.61 to 0.61) | 0.97 (0.96 to 0.98) | 0.47 (0.47 to 0.47) | 0.95 (0.94 to 0.96) | 0.99 (0.98 to 1.01) | 0.66 (0.65 to 0.67) | 0.46 (0.45 to 0.47) | 0.40 (0.39 to 0.41) | 0.50 (0.49 to 0.51) | 0.55 (0.54 to 0.56) | 0.36 (0.36 to 0.37) |
| Microscopic haematuria | 0.60 (0.58 to 0.62) | 0.93 (0.88 to 0.99) | 0.50 (0.48 to 0.52) | 1.07 (0.99 to 1.16) | 0.79 (0.73 to 0.86) | 0.34 (0.30 to 0.38) | 0.53 (0.48 to 0.59) | 0.42 (0.38 to 0.46) | 0.47 (0.42 to 0.52) | 0.51 (0.46 to 0.56) | 0.65 (0.60 to 0.69) |
| Lymphadenopathy | 0.44 (0.43 to 0.45) | 0.79 (0.77 to 0.82) | 0.31 (0.30 to 0.31) | 0.87 (0.84 to 0.90) | 0.71 (0.68 to 0.74) | 0.22 (0.21 to 0.24) | 0.28 (0.26 to 0.30) | 0.30 (0.29 to 0.32) | 0.39 (0.37 to 0.42) | 0.35 (0.34 to 0.38) | 0.30 (0.29 to 0.32) |
| Lymphadenopathy (neck) | 0.35 (0.34 to 0.35) | 0.75 (0.72 to 0.77) | 0.18 (0.17 to 0.18) | 0.80 (0.77 to 0.84) | 0.68 (0.65 to 0.72) | 0.11 (0.09 to 0.12) | 0.16 (0.14 to 0.17) | 0.19 (0.17 to 0.21) | 0.25 (0.23 to 0.28) | 0.22 (0.20 to 0.24) | 0.17 (0.16 to 0.18) |

**Supplementary Figures**

**SF1: Clinical feature consultation rates by week, cumulative clinical feature reports per week, and cumulative 2WW cancer referrals per week.** The dotted vertical lines denote the date of the first and second national lockdown.

**SF1a: Female cancer sites.**


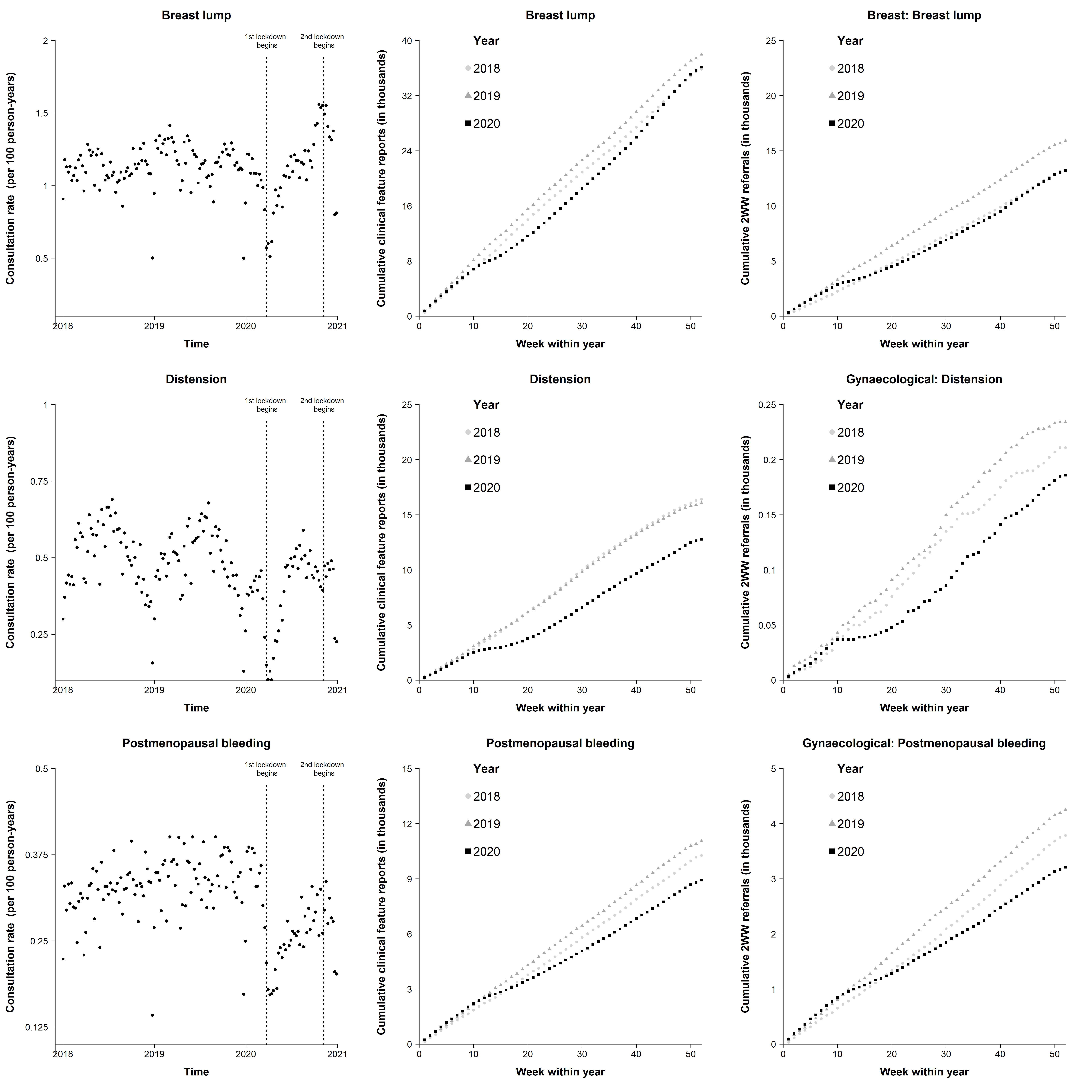


**SF1b: Colorectal cancer.**





**SF1c: Haematological and Head & Neck.**


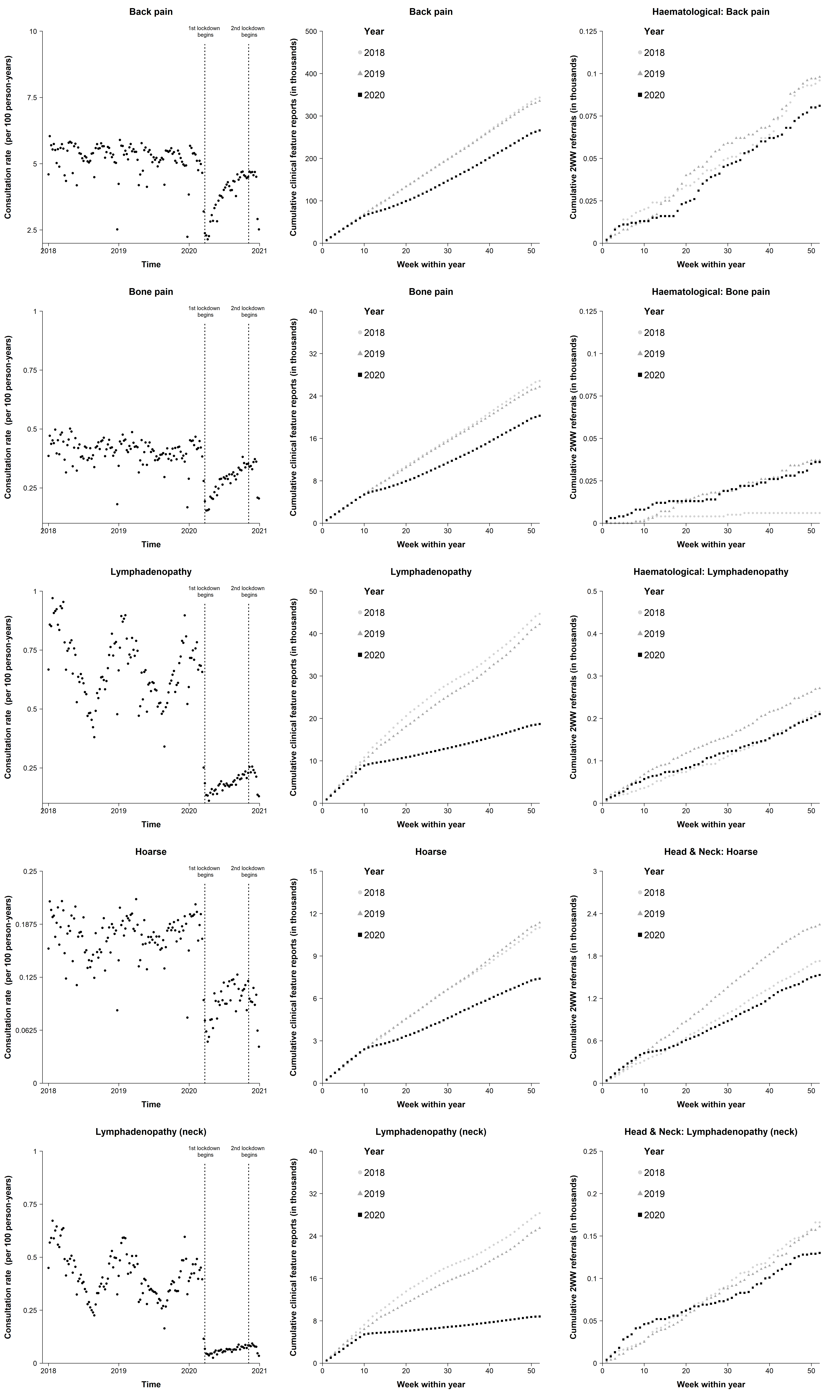


**SF1d: Lung cancer.**





**SF1e: Upper GI.**





**SF1f: Urological cancer.**


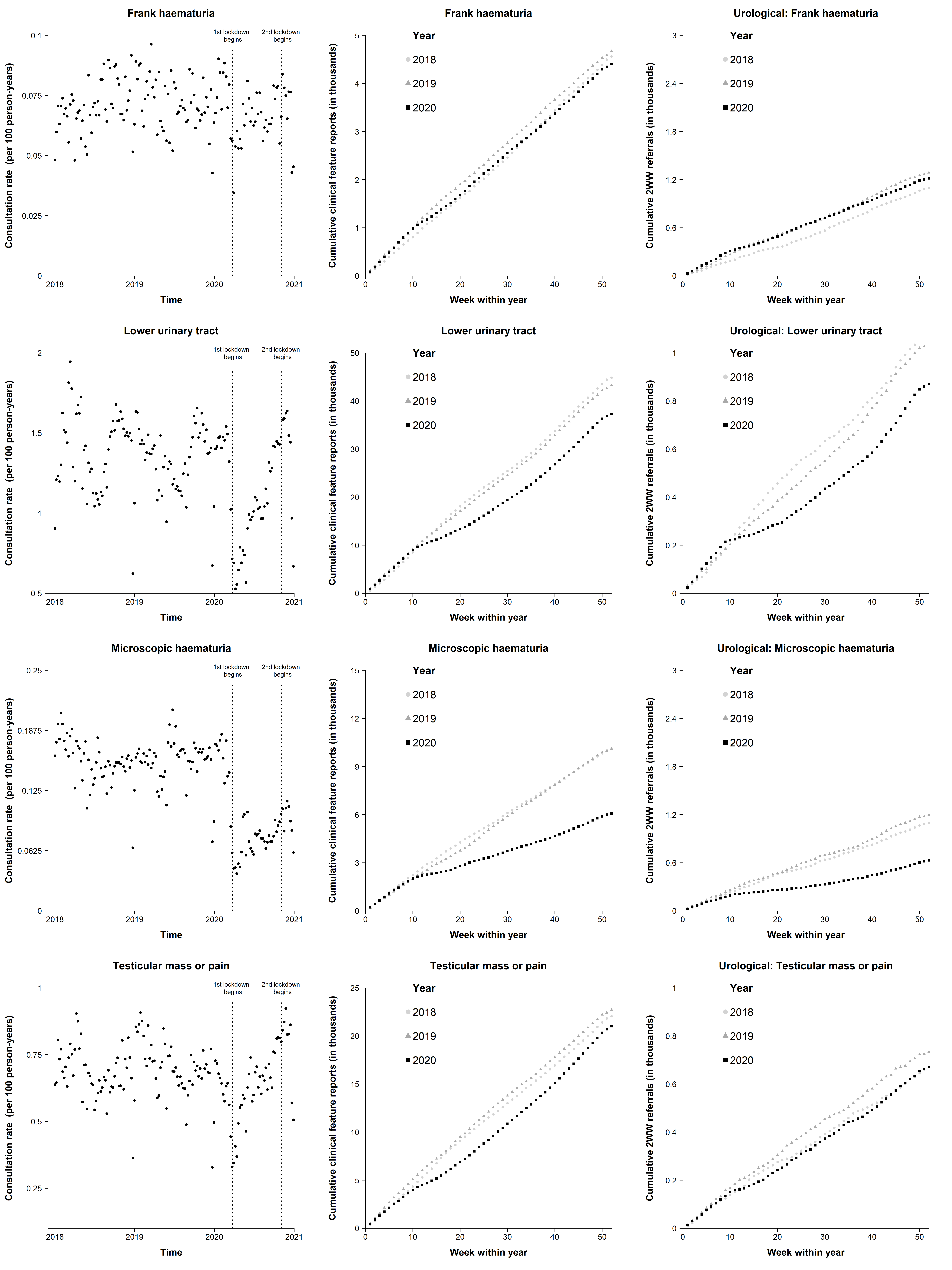


**SF2 – Overall weekly referral rates for 2WW pathways.**


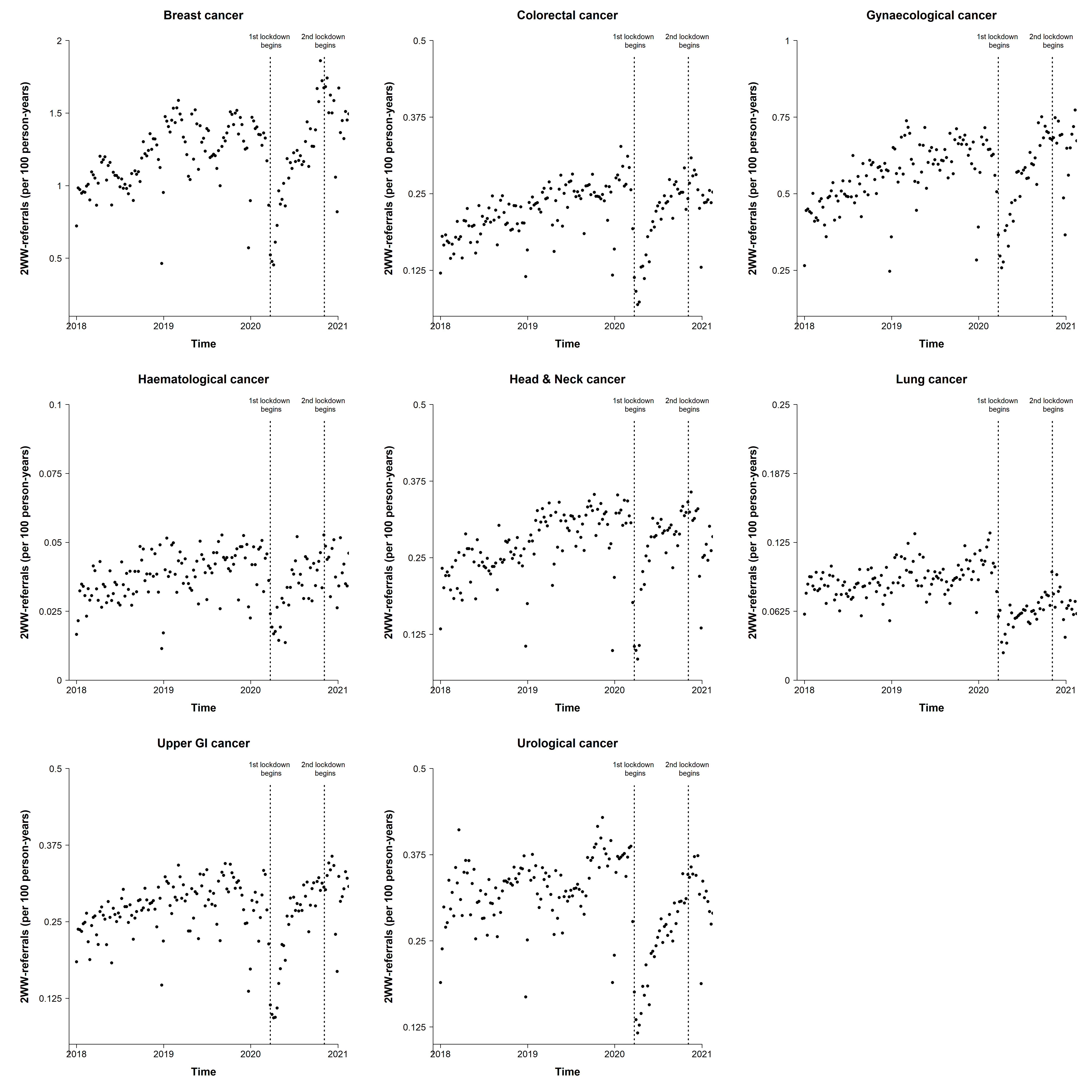

Supplement: Supplementary file 1 — Supplemental Material - Supplemental tables and figures [file 41416_2021_1666_MOESM1_ESM.docx]
