# Supplementary material for: Consultations for clinical features of possible cancer and associated urgent referrals before and during the COVID-19 pandemic: an observational cohort study from English primary care
Source: Br J Cancer. 2021 Dec 21;126(6):948–56. doi: 10.1038/s41416-021-01666-6 (PMC8691390; doi:10.1038/s41416-021-01666-6)
Supplement: Supplementary file 2 — RECORD checklist [file 41416_2021_1666_MOESM2_ESM.docx]

**The RECORD statement – checklist of items, extended from the STROBE statement, that should be reported in observational studies using routinely collected health data.**

|  | **Item**  **No.** | **STROBE items** | **Location in**  **manuscript where items are reported** | **RECORD items** | **Location in**  **manuscript**  **where items are reported** |
| --- | --- | --- | --- | --- | --- |
| **Title and abstract** | | | | | |
|  | 1 | (a) Indicate the study’s design with a commonly used term in the title or the abstract (b)  Provide in the abstract an  informative and balanced  summary of what was done and what was found | Page 1 (title)  Page 2 (abstract) | RECORD 1.1: The type of data used should be specified in the title or abstract. When possible, the name of the databases used should be included.  RECORD 1.2: If applicable, the geographic region and timeframe within which the study took place should be reported in the title or abstract.  RECORD 1.3: If linkage between databases was conducted for the study, this should be clearly stated in the title or abstract. | Page 2 (abstract, under Setting paragraph)  Page 2 (abstract, under Participants paragraph)  N/A |
| **Introduction** | | | | | |
| Background  rationale | 2 | Explain the scientific  background and rationale for the investigation being reported | Page 3 (Introduction section, paragraphs 1 and 2) |  |  |
| Objectives | 3 | State specific objectives,  including any prespecified hypotheses | Page 4 (Introduction section, last paragraph) |  |  |
| **Methods** | | | | | |
| Study Design | 4 | Present key elements of study design early in the paper | Page 5 (Methods section, under Study design and population subsection, first paragraph) |  |  |
| Setting | 5 | Describe the setting, locations, and relevant dates, including periods of recruitment, exposure, follow-up, and data collection | Page 5 (Methods section, under Study design and population subsection, first paragraph) |  |  |

| Participants | 6 | *(a) Cohort study*- Give the eligibility criteria, and the  sources and methods of selection of participants. Describe  methods of follow-up  *Case-control study*- Give the eligibility criteria, and the  sources and methods of case ascertainment and control  selection. Give the rationale for the choice of cases and controls *Cross-sectional study*- Give the eligibility criteria, and the  sources and methods of selection of participants  *(b) Cohort study*- For matched studies, give matching criteria and number of exposed and unexposed  *Case-control study*- For  matched studies, give matching criteria and the number of  controls per case | Page 5 (Methods section, under Study design and population subsection, first paragraph)  N/A | RECORD 6.1: The methods of study population selection (such as codes or algorithms used to identify subjects) should be listed in detail. If this is not possible, an explanation should be provided.  RECORD 6.2: Any validation studies of the codes or algorithms used to select the population should be referenced. If validation was conducted for this study and not published elsewhere, detailed methods and results should be provided.  RECORD 6.3: If the study involved linkage of databases, consider use of a flow diagram or other graphical display to demonstrate the data linkage process, including the number of individuals with linked data at each stage. | See appendix, files called "*Simplified SNOMED CT codes for clinical features.csv*" and "*Simplified SNOMED CT codes for cancer referral.csv*"  N/A  N/A |
| --- | --- | --- | --- | --- | --- |
| Variables | 7 | Clearly define all outcomes, exposures, predictors, potential confounders, and effect modifiers. Give diagnostic criteria, if applicable. | Page 5 (Methods section, under Clinical features and urgent referrals subsection) | RECORD 7.1: A complete list of codes and algorithms used to classify exposures, outcomes, confounders, and effect modifiers should be provided. If these cannot be reported, an explanation should be provided. | See above |
| Data sources/  measurement | 8 | For each variable of interest, give sources of data and details of methods of assessment (measurement).  Describe comparability of  assessment methods if there is more than one group | N/A |  |  |
| Bias | 9 | Describe any efforts to address potential sources of bias | Regarding standardisation, see pages 5-6 (Methods section, under Statistical analysis subsection, second paragraph). |  |  |
| Study size | 10 | Explain how the study size was arrived at | The whole dataset was used. The sample size is described in page 7 (Results section, first paragraph). |  |  |
| Quantitative  variables | 11 | Explain how quantitative  variables were handled in the analyses. If applicable, describe which groupings were chosen, and why | N/A |  |  |
| Statistical  methods | 12 | (a) Describe all statistical  methods, including those used to control for confounding  (b) Describe any methods used to examine subgroups and interactions  (c) Explain how missing data were addressed  (d) *Cohort study*- If applicable, explain how loss to follow-up was addressed  *Case-control study*- If  applicable, explain how  matching of cases and controls was addressed  *Cross-sectional study*- If  applicable, describe analytical methods taking account of sampling strategy  (e) Describe any sensitivity analyses | Pages 5-6 (Methods section, under Statistical analysis subsection)  N/A  N/A  N/A  N/A |  |  |
| Data access and cleaning methods |  | .. |  | RECORD 12.1: Authors should describe the extent to which the investigators had access to the database population used to create the study population. | N/R  We are happy to provide these details upon request. |
|  |  |  |  | RECORD 12.2: Authors should provide information on the data cleaning methods used in the study. | N/R  We are happy to provide these details upon request. |
| Linkage |  | .. |  | RECORD 12.3: State whether the study included person-level, Institutional-level, or other data linkage across two or more databases. The methods of linkage and methods of linkage quality evaluation should be provided. | N/A |

| **Results** | | | | | |
| --- | --- | --- | --- | --- | --- |
| Participants | 13 | (a) Report the numbers of  individuals at each stage of the study (*e.g.*, numbers potentially eligible, examined for eligibility, confirmed eligible, included in the study, completing follow-up, and analysed)  (b) Give reasons for non-  participation at each stage.  (c) Consider use of a flow  diagram | The whole dataset was used. The sample size is described in page 7 (Results section, first paragraph).  N/A  N/A | RECORD 13.1: Describe in detail the selection of the persons included in the study (*i.e.,* study population selection) including filtering based on data quality, data availability and linkage. The selection of included persons can be described in the text and/or by means of the study flow diagram. | Page 5 (Methods section, under Clinical features and urgent referrals subsection). We are happy to provide more detail upon request. |
| Descriptive data | 14 | (a) Give characteristics of study participants (*e.g.*, demographic, clinical, social) and information on exposures and potential  confounders  (b) Indicate the number of participants with missing data for each variable of interest (c) *Cohort study*- summarise follow-up time (*e.g.*, average and total amount) | Page 7 (Results section, under Cohort description).  N/A  N/A |  |  |
| Outcome data | 15 | *Cohort study*- Report numbers of outcome events or summary measures over time  *Case-control study*- Report numbers in each exposure category, or summary measures of exposure  *Cross-sectional study*- Report numbers of outcome events or summary measures | Page 7 (Results section, under Clinical feature consultation rate ratios subsection, last lines) |  |  |

| Main results | 16 | (a) Give unadjusted estimates and, if applicable, confounder- adjusted estimates and their precision (e.g., 95% confidence interval). Make clear which confounders were adjusted for and why they were included  (b) Report category boundaries when continuous variables were categorized  (c) If relevant, consider  translating estimates of relative risk into absolute risk for a meaningful time period | Figures 2 and 3  ST1  Page 6 (Methods section, under Statistical analysis subsection, 4^th^ paragraph, categorization of year into 6 week periods)  Figure 3 |  |  |
| --- | --- | --- | --- | --- | --- |
| Other analyses | 17 | Report other analyses done— e.g., analyses of subgroups and interactions, and sensitivity analyses | N/A |  |  |
| **Discussion** | | | | | |
| Key results | 18 | Summarise key results with reference to study objectives | Page 9 (Discussion section, under Summary of main findings subsection). |  |  |
| Limitations | 19 | Discuss limitations of the study, taking into account sources of potential bias or imprecision. Discuss both direction and magnitude of any potential bias | Pages 9-10 (Discussion section, under Strengths and limitations subsection). | RECORD 19.1: Discuss the implications of using data that were not created or collected to answer the specific research question(s). Include discussion of misclassification bias, unmeasured confounding, missing data, and changing eligibility over time, as they pertain to the study being reported. | Pages 9-10 (Discussion section, under Strengths and limitations subsection). |
| Interpretation | 20 | Give a cautious overall  interpretation of results  considering objectives, limitations, multiplicity of analyses, results from similar studies, and other relevant evidence | Page 13 (Conclusion section) |  |  |
| Generalisability | 21 | Discuss the generalisability (external validity) of the study results | N/R |  |  |

| **Other Information** | | | | | |
| --- | --- | --- | --- | --- | --- |
| Funding | 22 | Give the source of funding and the role of the funders for the present study and, if applicable, for the original study on which the present article is based | Page 14 (Funding section) |  |  |
| Accessibility of protocol, raw  data, and  programming  code |  | .. |  | RECORD 22.1: Authors should provide information on how to access any supplemental information such as the study protocol, raw data, or programming code. | We are happy to provide this upon request. |

*Reference: Benchimol EI, Smeeth L, Guttmann A, Harron K, Moher D, Petersen I, Sørensen HT, von Elm E, Langan SM, the RECORD Working Committee. The REporting of studies Conducted using Observational Routinely-collected health Data (RECORD) Statement. *PLoS Medicine* 2015; in press.

*Checklist is protected under Creative Commons Attribution (CC BY) license.
